# Single-cell dissection of self-derived versus donor-derived T cell exhaustion niches in patients with acute myeloid leukemia

**DOI:** 10.1016/j.isci.2026.117918

**Published:** 2026-10-16

**Authors:** Chun-Zi Yu, Yu-Lun Chen, Xiao-Hui Zhang, Lan-Ping Xu, Yu Wang, Huan Chen, Yu-Hong Chen, Feng-Rong Wang, Yu-Qian Sun, Yuan-Yuan Zhang, Meng Lv, Xiao-Dong Mo, Yi-Fei Cheng, Kai-Yan Liu, Xiao-Su Zhao, Xiao-Qiang Sun, Xiang-Yu Zhao, Xiao-Jun Huang, Ying-Jun Chang

**Affiliations:** 1 Peking University People’s Hospital & Peking University Institute of Hematology, National Clinical Research Center for Hematologic Disease, Beijing Key Laboratory of Hematopoietic Stem Cell Transplantation, No. 11 South Street of Xizhimen, Xicheng District, Beijing 100044, China; 2 Peking-Tsinghua Center for Life Sciences, Beijing 100871, China; 3 Research Unit of Key Technique for Diagnosis and Treatments of Hematologic Malignancies, Chinese Academy of Medical Sciences, 2019RU029, Beijing, China; 4 Zhong-Shan School of Medicine, Sun Yat-Sen University, Guangzhou 510080, China

**Keywords:** acute myeloid leukemia, T cell exhaustion, single-cell atlas, immune microenvironment

## Abstract

Relapse remains the main cause of death in acute myeloid leukemia (AML), and T cell exhaustion is related to relapse in both non-allografting and allografting settings. However, differences in exhausted T cells (T_ex_) across different disease contexts remain incompletely understood. We performed single-cell RNA and T cell receptor (TCR) sequencing to systematically characterize T_ex_ in AML. Both self-derived T_ex_ in non-transplantation and donor-derived T_ex_ in post-transplant settings express similar surface markers, but NFATC1/NFATC3 with downstream TOX and EOMES are enriched in self-derived T_ex_, while NFATC2 with downstream NR4A2 predominates in donor-derived T_ex_. Both self-derived and donor-derived bone marrow environments are enriched with immunosuppressive monocytes, dendritic cells, and regulatory T cells. Cell-cell interaction analysis reveals strong direct and indirect interactions between T_ex_ and AML cells, primarily mediated by galectin-9, MIF, and TGF-β1. These findings highlight differences and commonalities between self-derived and donor-derived T_ex_ and identify potential therapeutic targets for reversing T_ex_ in AML.

## Highlights

• Multi-omic analysis reveals immunosuppressive BM microenvironments pre/post-transplant in AML
• Distinct NFAT-centered transcriptional programs distinguish self- and donor-derived CD8^+^ T_ex_
• Immunosuppressive monocytes, DCs, and Tregs accumulate in self- and donor-derived BM
• AML cells interact directly and indirectly with CD8^+^ T_ex_ via galectin-9, MIF, and TGF-β1

## Graphical abstract

**Figure:**
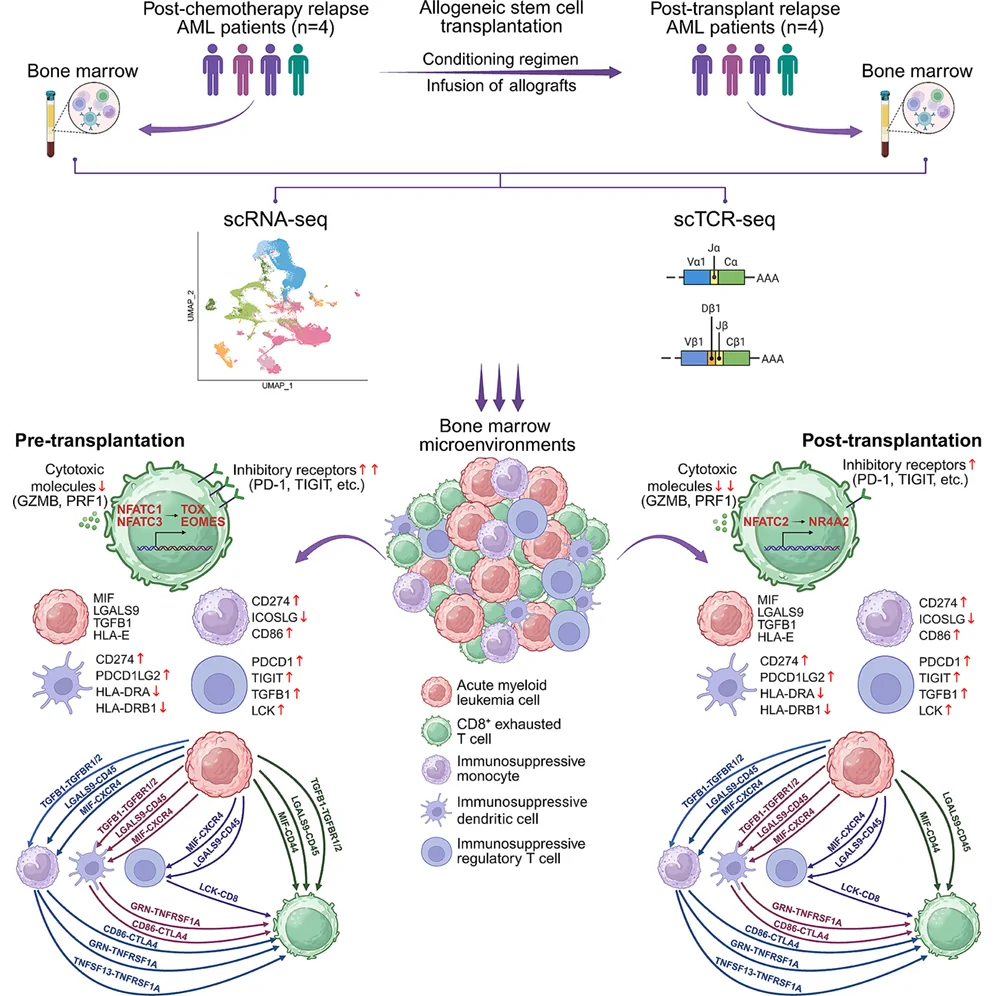

## Teaser

Immunology; Microenvironment

## Introduction

Acute myeloid leukemia (AML) is a heterogeneous malignant clonal disorder that seriously endangers human health.^1^ Although the clinical use of targeted therapy and allogeneic stem cell transplantation (allo-SCT) has improved the outcomes of AML patients, most patients eventually relapse, with a 5-year survival rate of 30%.^2^^,^^3^

Cytotoxic CD8^+^ T cells are effector cells and form the backbone of cancer immunotherapy.^4^ Under conditions of chronic inflammation and tumors, if the elimination of target cells fails and T cell receptor (TCR) signaling is sustained, T cells may become “exhausted”, which is characterized by elevated co-expression of inhibitory receptors (IRs) such as PD-1, TIM-3, LAG-3, and CTLA-4.^5^ T cell exhaustion may not only be related to relapsed or refractory (R/R) disease in chemotherapy and targeted therapy settings (self-derived exhausted T cells, T_ex_) but also contribute to leukemia relapse in allo-SCT settings (donor-derived T_ex_) for patients with AML.^6^^,^^7^^,^^8^ Although CD8^+^ T_ex_ and immunosuppressive myeloid populations have previously been described in AML,^6^^,^^7^^,^^8^^,^^9^^,^^10^ the similarities and differences between self-derived and donor-derived T_ex_ populations, particularly with respect to their transcriptional programs and interactions with leukemia cells, remain poorly understood.

Technological developments in single-cell RNA sequencing (scRNA-seq) and immune profiling have allowed for the comprehensive analysis of tumor microenvironments at the transcript and TCR levels, facilitating the investigation of cellular phenotypes and molecular regulation.^11^^,^^12^ In addition, scRNA-seq is employed to explore the intercellular interactions associated with T cell exhaustion within the tumor microenvironment, with the goal of identifying potential targets for immunotherapy.^13^^,^^14^ To our knowledge, no previous study has used scRNA-seq to systematically compare the exhaustion-associated regulatory programs and cellular communication networks between leukemia cells and self-derived versus donor-derived CD8^+^ T_ex_ in R/R AML.

Using an allo-SCT platform combined with scRNA-seq and scTCR-seq, we systematically characterized CD8^+^ T cell exhaustion in the bone marrow (BM) of R/R AML patients. We identified distinct transcriptional networks in self-derived (pre-transplant) versus donor-derived (post-transplant) T_ex_: self-derived T_ex_ were characterized by enrichment of NFATC1/NFATC3-, TOX-, and EOMES-associated transcriptional programs, whereas donor-derived T_ex_ were associated with NFATC2- and NR4A2-related regulatory signatures. Both populations resided in an immunosuppressive microenvironment enriched with monocytes, dendritic cells (DCs), and regulatory T cells (Tregs). Strong leukemic T cell interactions, mediated directly or indirectly via galectin-9, MIF, and TGF-β1, were observed. Our study identifies TOX, NR4A2, galectin-9, MIF, and TGF-β1 as potential therapeutic targets to alleviate T cell exhaustion in AML.

## Results

### Comprehensive single-cell atlas of immune cell and leukemia-like cell compartments in AML patients before and after transplantation

Four AML patients were traced at two disease stages: pre-SCT nonremission (NR) (group 1) and post-SCT relapse (group 2) (Figures 1A and S1A and Data S1). We derived graph-based clusters composed of 95,426 cells for cell type annotation (Figure S1B). We first analyzed each single sample from each AML patient in combination with four healthy donors (HDs) independently (see STAR Methods and Figures S1C–S1I); the integrated results are shown in Figures 1B and 1C. This analysis revealed 24,463 T cells, 14,709 cells of myeloid lineage (monocytes, DCs, and so forth), and 37,134 leukemia-like cells. We observed that the frequencies of T cells in the BM of both groups 1 and 2 were significantly lower than those in the HDs (Figures 1D and S2A).

**Figure 1:**
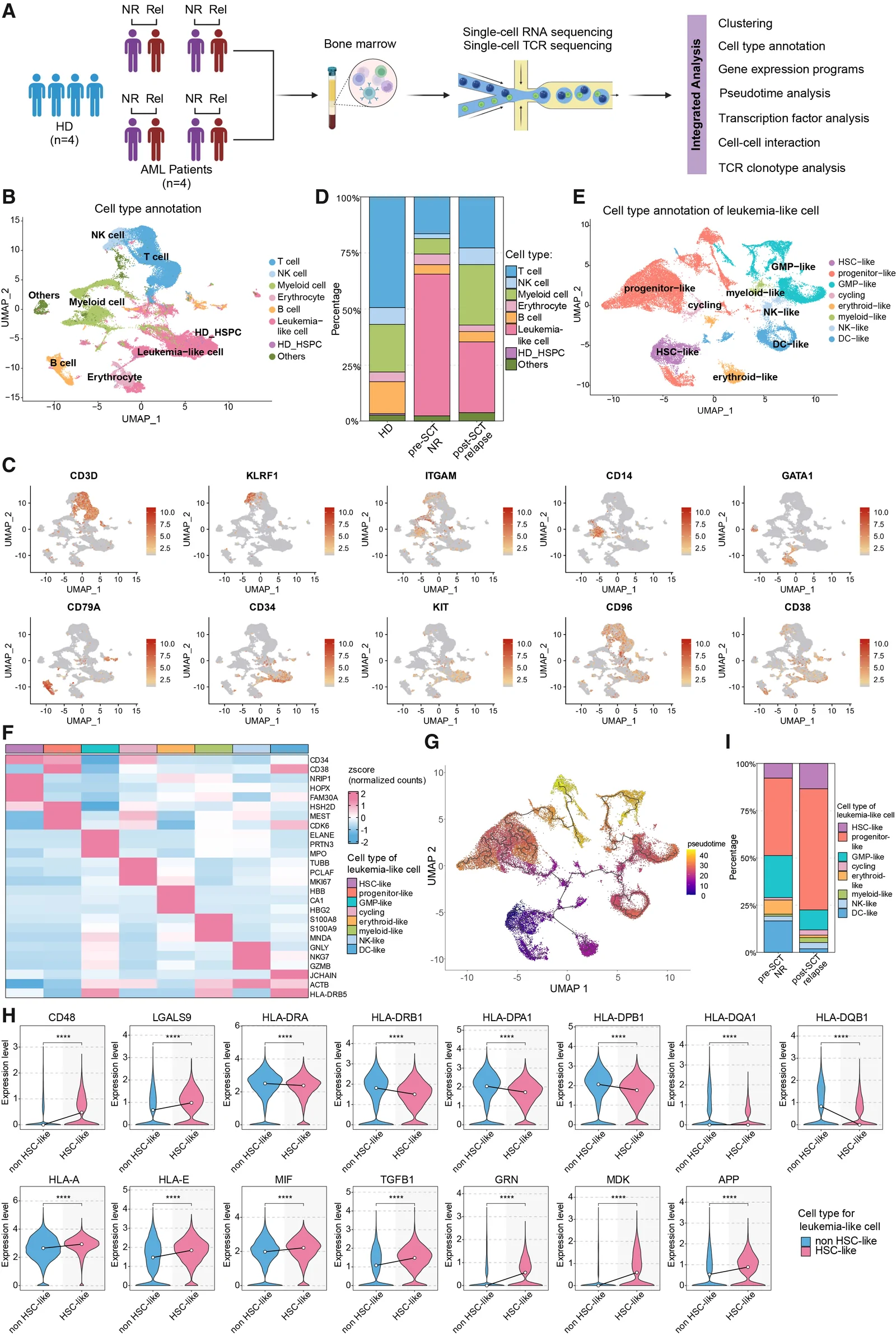
Single-cell transcriptomic landscape of bone marrow environments in AML patients and characteristics of leukemia-like cells (A) Sample composition (four AML patients who were traced at both pre-SCT NR stage and post-SCT relapse stage, and four HDs) and experimental approach. (B) Uniform manifold approximation and projection (UMAP) plot of scRNA-seq data from all 95,426 cells colored by cell type. (C) UMAP plot showing transcriptional expression distribution of main cell type markers. (D) Proportion of different cell types in HD, pre-SCT NR and post-SCT relapse sample types. (E) UMAP plot of all 37,134 leukemia-like cells colored by leukemia-like cell type. (F) Heatmap showing normalized average expression of selected marker genes for leukemia-like cell subpopulation. (G) Pseudotime trajectory (black lines) and pseudotime (color gradient) for leukemia-like cells projected on UMAP embedding. Trajectory root is set from the white dot. (H) Violin plot showing the expression levels of selected marker genes, comparing HSC-like blasts with non-HSC-like blasts. The white dot inside each violin plot represented the median. ∗∗∗∗*p* < 0.0001. Wilcoxon rank-sum test (two-sided). (I) Proportion of different leukemia-like cell subtypes in pre-SCT NR and post-SCT relapse sample types. Abbreviations: HD, healthy donor; NR, pre-stem-cell transplantation nonremission; Rel, post-stem-cell transplantation relapse; AML, acute myeloid leukemia; TCR, T cell receptor.

On the basis of the previous research^15^^,^^16^^,^^17^ and marker gene analysis (Figure S2B and Data S2), we classified leukemia cells into hematopoietic stem cell (HSC)-like blasts, progenitor-like blasts, granulocyte-monocyte progenitor (GMP)-like blasts, actively cycling blasts, erythroid-like blasts, differentiating myeloid-like blasts, natural killer (NK)-like blasts, and DC-like blasts (Figures 1E and 1F). The HSC-like blasts had the highest leukemic stem cell 17-gene (LSC17) score^18^ (Figure S2C). Pseudotime analysis revealed a trajectory from HSC-like blasts to other subtypes of blasts, supporting a continuum of cell differentiation (Figure 1G). Differentially expressed gene (DEG) analysis and Gene Ontology (GO) enrichment analysis revealed that compared with those in non-HSC-like blasts, the upregulated pathways in HSC-like blasts included leukocyte proliferation, regulation of hemopoiesis, and negative regulation of immune system processes (Figure S2D and Data S3). Previous studies have shown that the secretion or expression of molecules such as MIF, TGFB1, LGALS9, and HLA-E by tumor cells can promote the exhaustion of T cells or NK cells.^19^^,^^20^^,^^21^^,^^22^ We found that compared with non-HSC-like blasts, HSC-like blasts upregulated the expression levels of *MIF*, *TGFB1*, *LGALS9*, and *HLA-E* and downregulated the expression levels of HLA class II molecules (Figure 1H). The proportions of HSC-like blasts and progenitor-like blasts in the BM of group 2 were greater than those in group 1 (Figure 1I), and the LSC17 score for HSC-like blasts was significantly greater in group 2 than in group 1 (Figure S2E). These findings suggest that HSC-like blasts with enhanced stemness and immunosuppressive capacity might result in post-SCT relapse. Given the reported role of HLA class II downregulation in post-SCT immune escape,^23^ we compared HLA class II expression in HSC-like blasts between groups 1 and group 2. Notably, expression patterns were heterogeneous, with HLA-DR being upregulated, while HLA-DP and HLA-DQ being downregulated after transplantation (Figure S2F). These findings indicate that antigen-presentation-related alterations in group 2 may be molecule-specific rather than uniformly suppressed.

### T_ex_ clusters identified in transplantation recipients with AML

The annotated T cell clusters included two naive clusters (CD4^+^ and CD8^+^), two memory clusters (CD4^+^ and CD8^+^), and three effector clusters (CD4^+^, CD8^+^, and NKT) (Figures 2A and 2B).^14^^,^^24^ T cell subtypes also include Tregs, mucosal-associated invariant T cells, and γδ T cells.^24^ In addition, three T cell clusters were characterized by high expression of IRs (such as *PDCD1*, *LAG3*, and *TIGIT*), which we defined as IR^high^ T cell clusters (CD8^+^, CD4^+^, and NKT) (Figure 2B).^24^^,^^25^ We found that the proportions of CD8^+^ effector T cells (Teff) and CD8^+^ IR^high^ T cells in the BM of groups 1 and 2 were greater than those in the BM of HDs (Figures 2C and S3A).

**Figure 2:**
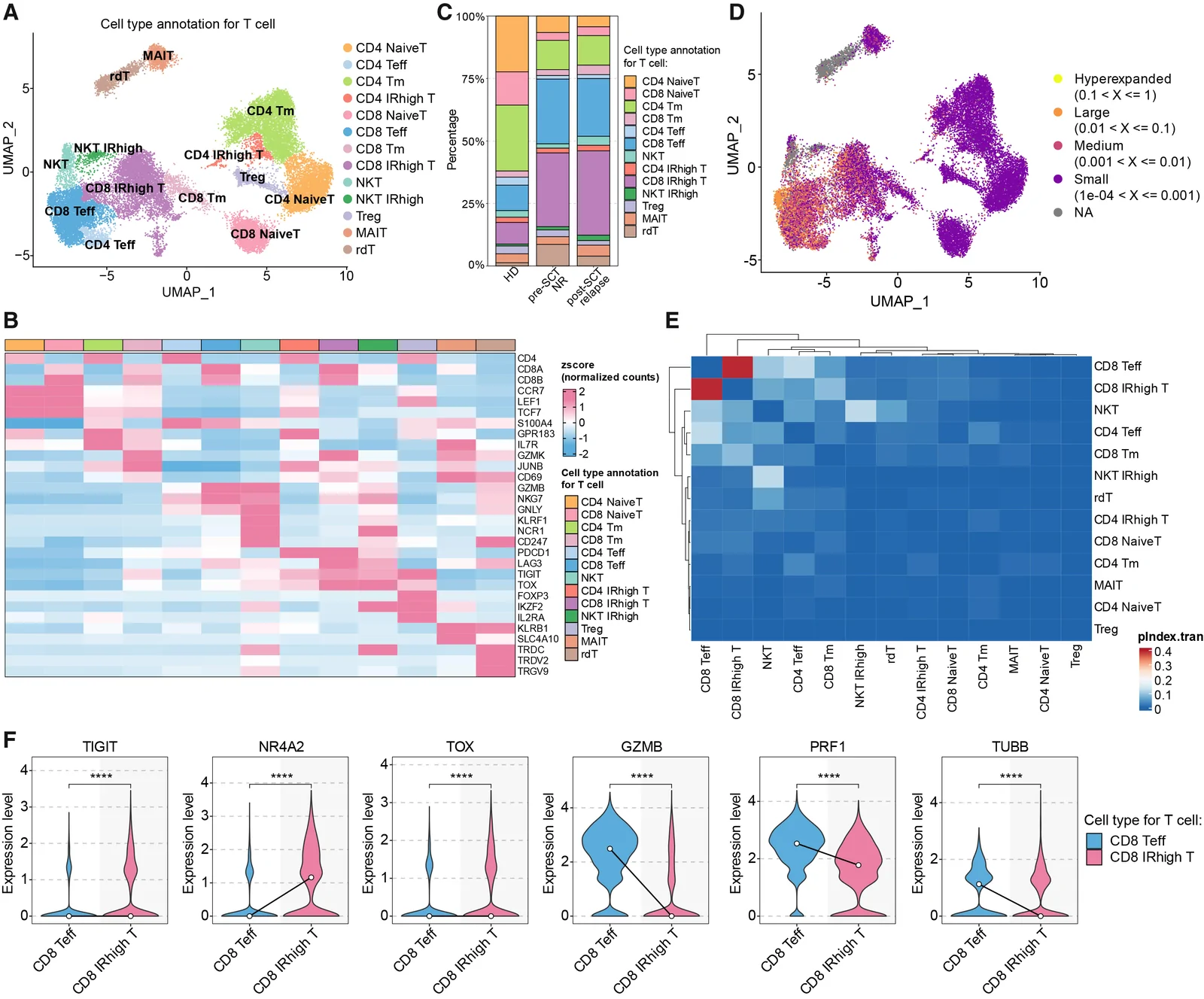
The T cell landscape of bone marrow in AML patients (A) UMAP plot of all 24,463 T cells colored by T cell types. (B) Heatmap showing normalized average expression of selected marker genes for different T cell types. (C) Proportion of different T cell subtypes in HD, pre-SCT NR and post-SCT relapse sample types. (D) UMAP plot showing the clonal expansion status of T cells from AML patients. The colors corresponded to four TCR clonal expansion groups. (E) Heatmap visualization of pairwise transition likelihood (based on clonotypes) of T cell subtypes. (F) Violin plot showing the expression levels of selected marker genes representing exhaustion, cytotoxicity, and proliferation in CD8^+^ Teff cells compared to CD8^+^ IR^high^ T cells. The white dot inside each violin plot represented the median. ∗∗∗∗*p* < 0.0001. Wilcoxon rank-sum test (two-sided).

Clonal analysis of T cells^24^ revealed that highly expanded cell subsets were concentrated mainly in CD8^+^ Teff and CD8^+^ IR^high^ clusters in the BM of both groups 1 and 2, whereas the population with limited clonal expansion was mostly focused in the naive and memory clusters in the HD sample type (Figures 2D, S3B, and S3C). There was a strong correlation between CD8^+^ Teff and CD8^+^ IR^high^ T cells (Figures S3D and S3E). The calculated pairwise transition index (pIndex.tran)^26^ revealed that CD8^+^ Teff and CD8^+^ IR^high^ T cells had the highest pairwise transition likelihood (Figure 2E). These results suggest the transition potential of IR^high^ T cells from Teff.

In quantitative analysis, compared with CD8^+^ Teff, CD8^+^ IR^high^ T cells expressed lower levels of *GZMB*, *PRF1*, and *TUBB* but higher levels of IRs (e.g., *TIGIT*) and transcription factors (TFs) (e.g., *TOX* and *NR4A2*)^24^^,^^27^ (Figures 2F and S3F). Similar results were observed in the comparisons between CD4^+^ effector cells and CD4^+^ IR^high^ T cells and between NKT cells and NKT IR^high^ cells (Figures S3G and S3H). Compared with those of CD8^+^ Teff, the exhaustion score of CD8^+^ IR^high^ T cells was significantly greater, while the cytotoxic score and proliferation score were significantly lower (Figure S3I and Data S4).^24^^,^^25^^,^^28^ These results indicate the presence of T_ex_ in the BM of both groups 1 and 2.

### Differential gene expression programs in T_ex_ clusters between self-derived and donor-derived T cells

We subsequently found that the medium and large clonal index clonotypes occupied more repertoire space in group 2 than in group 1 (Figure S4A). With respect to gene expression, for CD8^+^ T_ex_, the expression levels of most IRs and effector molecules (*GZMB* and *PRF1*) were significantly greater in group 1 than those in group 2 (Figures 3A and S4B). The exhaustion and cytotoxicity scores were greater in group 1, whereas the proliferation score was greater in group 2 (Figure S4C). The expression levels of exhaustion-related TFs were higher for TOX and EOMES in group 1 but higher for NR4A2 in group 2 (Figure 3A). Although the patient-level analysis did not reach statistical significance, most patients exhibited consistent trends in TFs expression changes (Figure S4D), suggesting that the differences observed at the single-cell level may reflect an overall shift in exhaustion-associated transcriptional programs. These results indicate that distinct TF-associated regulatory programs are enriched in self-derived (group 1) and donor-derived (group 2) T_ex_ populations.

**Figure 3:**
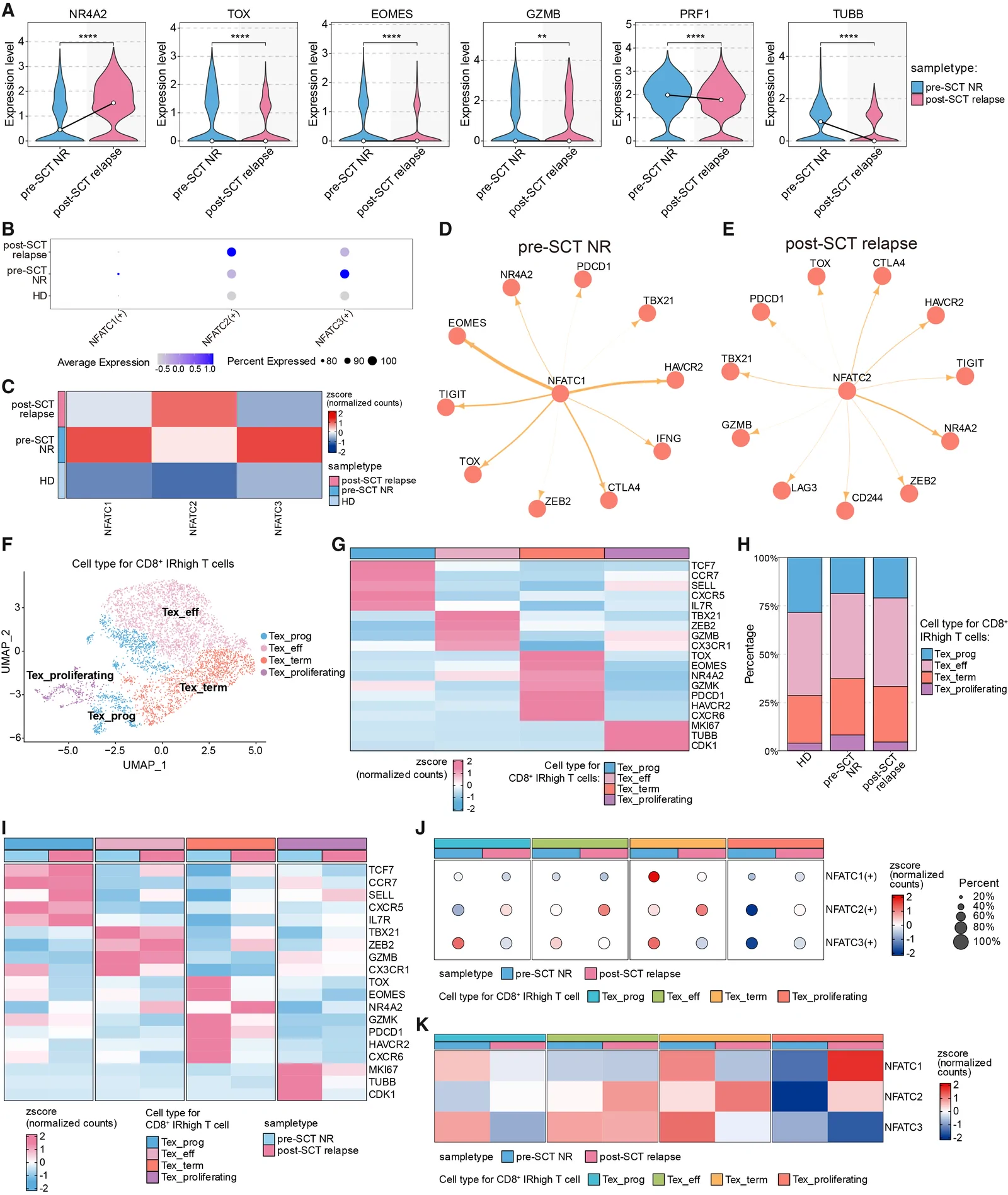
The CD8^+^ exhausted T cell landscape of bone marrow in AML patients (A) Violin plot showing the expression levels of selected marker genes representing exhaustion, cytotoxicity, and proliferation in CD8^+^ T_ex_, comparing pre-SCT NR and post-SCT relapse sample types. The white dot inside each violin plot represented the median. ∗∗*p* < 0.01 and ∗∗∗∗*p* < 0.0001. Wilcoxon rank-sum test (two-sided). (B) Dot plot showing activation of selected TF motifs evaluated by pySCENIC for HD, pre-SCT NR and post-SCT relapse sample types in CD8^+^ T_ex_. (C) Heatmap showing normalized average expression of selected TF motifs for HD, pre-SCT NR and post-SCT relapse sample types in CD8^+^ T_ex_. (D) Gene regulatory network of transcription factor NFATC1 for pre-SCT NR sample type in CD8^+^ T_ex_. (E) Gene regulatory network of transcription factor NFATC2 for post-SCT relapse sample type in CD8^+^ T_ex_. (F) UMAP plot of all 4,913 CD8^+^ T_ex_ colored by CD8^+^ T_ex_ subtypes. (G) Heatmap showing normalized average expression of selected marker genes for different CD8^+^ T_ex_ subtypes. (H) Proportion of different CD8^+^ T_ex_ subtypes in HD, pre-SCT NR and post-SCT relapse sample types. (I) Heatmap showing normalized average expression of selected marker genes for different CD8^+^ T_ex_ subtypes splited by different sample types. (J) Dot plot showing activation of selected TF motifs evaluated by pySCENIC for different CD8^+^ T_ex_ subtypes splited by different sample types. (K) Heatmap showing normalized average expression of selected TF motifs for different CD8^+^ T_ex_ subtypes splited by different sample types. For dot plots (B and J), the colors represent the normalized area under the curve (AUC) of selected TF motifs, and dot sizes represent the percentage activity of selected TF motifs in the indicated clusters. For gene regulatory network plots (D and E), the direction of the arrow represents the direction of gene regulation, and the thickness of the line represents the weight of regulation.

During chronic infection and cancer, persistent expression of IRs in CD8^+^ T cells disrupts the activation of c-Jun/AP-1, leading to partnerless NFAT that triggers exhaustion-related gene expression.^29^^,^^30^ NFAT family members, including NFATC1, NFATC2, and NFATC3, play important roles in T cell development and function.^30^^,^^31^ Although multiple TF-associated programs were identified in our analyses, we focused primarily on NFAT family members given the established roles of NFAT signaling in T cell exhaustion. In CD8^+^ T_ex_, NFATC1 and NFATC3 were highly activated and expressed in group 1, whereas NFATC2 was highly activated and expressed in group 2 (Figures 3B and 3C). Regulons evaluated and ranked by the regulon-specific score (RSS)^32^ also suggested that NFATC1 and NFATC3 were among the top 100 ranked TFs in group 1, whereas NFATC2 was among the top 100 ranked TFs in group 2 (Figures S4E and S4F). Moreover, in CD8^+^ T_ex_, the NFAT family, especially NFATC1, regulated mainly the expression of TOX, EOMES and multiple IRs in group 1; however, the NFAT family, primarily NFATC2, regulated mainly the expression of NR4A2 and various IRs in group 2 (Figures 3D, 3E, and S4G). In addition, TOX and EOMES strongly regulated IR expression in the CD8^+^ T_ex_ in group 1; however, NR4A2 primarily regulated the expression of IRs and effector-related molecules in the CD8^+^ T_ex_ in group 2 (Figure S4H). Taken together, these findings suggest that self-derived CD8^+^ T_ex_ is characterized by enrichment of NFATC1-associated transcriptional programs together with increased TOX and EOMES expression, whereas donor-derived CD8^+^ T_ex_ exhibits enrichment of NFATC2-associated signatures accompanied by elevated NR4A2 expression.

We identified four major clusters of CD8^+^ T_ex_,^33^ including a progenitor population (T_ex_^prog^), an effector population (T_ex_^eff^), a more terminally exhausted population (T_ex_^term^), and an actively dividing population (Figures 3F and 3G).^33^^,^^34^ There was no significant difference in the proportion of CD8^+^ T_ex_ subsets among the HDs, group 1, and group 2 (Figures 3H and S5A). Slingshot trajectory inference analysis^35^ revealed transitions from T_ex_^prog^ to the other three T_ex_ subtypes (Figure S5B). TCR repertoire analysis revealed that the small clonal index clonotypes occupied more repertoire space in the T_ex_^eff^ and T_ex_^term^ subtypes, whereas the medium clonal index clonotype occupied more repertoire space in the T_ex_^prog^ and proliferating T_ex_ subtypes (Figure S5C). We found that the T_ex_^term^ population had the highest exhaustion score, the T_ex_^eff^ population had the highest cytotoxicity score, and the proliferating T_ex_ population had the highest proliferation score (Figure S5D). Next, we observed that the highly activated TFs in the T_ex_^prog^, T_ex_^eff^, and T_ex_^term^ populations were NFATC3, NFATC2, and NFATC1, respectively (Figure S5E). Gene regulatory network analysis revealed that in T_ex_^prog^ cells, NFATC3 mainly regulated the expressions of *HAVCR2*, *EOMES*, *SELL*, and *TCF7*. In T_ex_^eff^ cells, NFATC2 mainly regulated the expressions of effector-related genes such as *TBX21*, *ZEB2*, and *GZMB*, whereas in T_ex_^term^ cells, NFATC1 mainly regulated the expressions of genes associated with terminal exhaustion such as *TOX*, *EOMES*, *CTLA4*, *HAVCR2*, and *PDCD1* (Figures S5F–S5H).

In addition, we found that each of the four T_ex_ subtypes had higher expression levels of TOX and EOMES in group 1 but a higher expression level of NR4A2 in group 2 (Figure 3I). Analysis of TF activity suggested that in group 1, NFATC3 was highly activated and expressed in the T_ex_^prog^ and T_ex_^eff^ subtypes and that NFATC1 and NFATC3 were highly activated and expressed in the T_ex_^term^ subtype, whereas in group 2, NFATC2 was highly activated and expressed in all three subpopulations of T_ex_^prog^, T_ex_^eff^, and T_ex_^term^ (Figures 3J and 3K). Overall, these results indicate that the T_ex_^prog^ subtype can differentiate into other T_ex_ subtypes through the activation of different TFs, whose degree of exhaustion, cytotoxic potential, and proliferation capacity differ.

### The immunosuppressive BM microenvironment in patients with AML before and after transplantation

Graph-based subclustering of the scRNA-seq profiles from the myeloid cell fraction revealed five clusters, which included GMPs, monocytes, DCs, neutrophils, and basophils (Figures 4A and 4B).^13^^,^^36^ There was no significant difference in the proportions of the main myeloid cell subsets among the HDs, group 1, and group 2 (Figures 4C and S6A).

**Figure 4:**
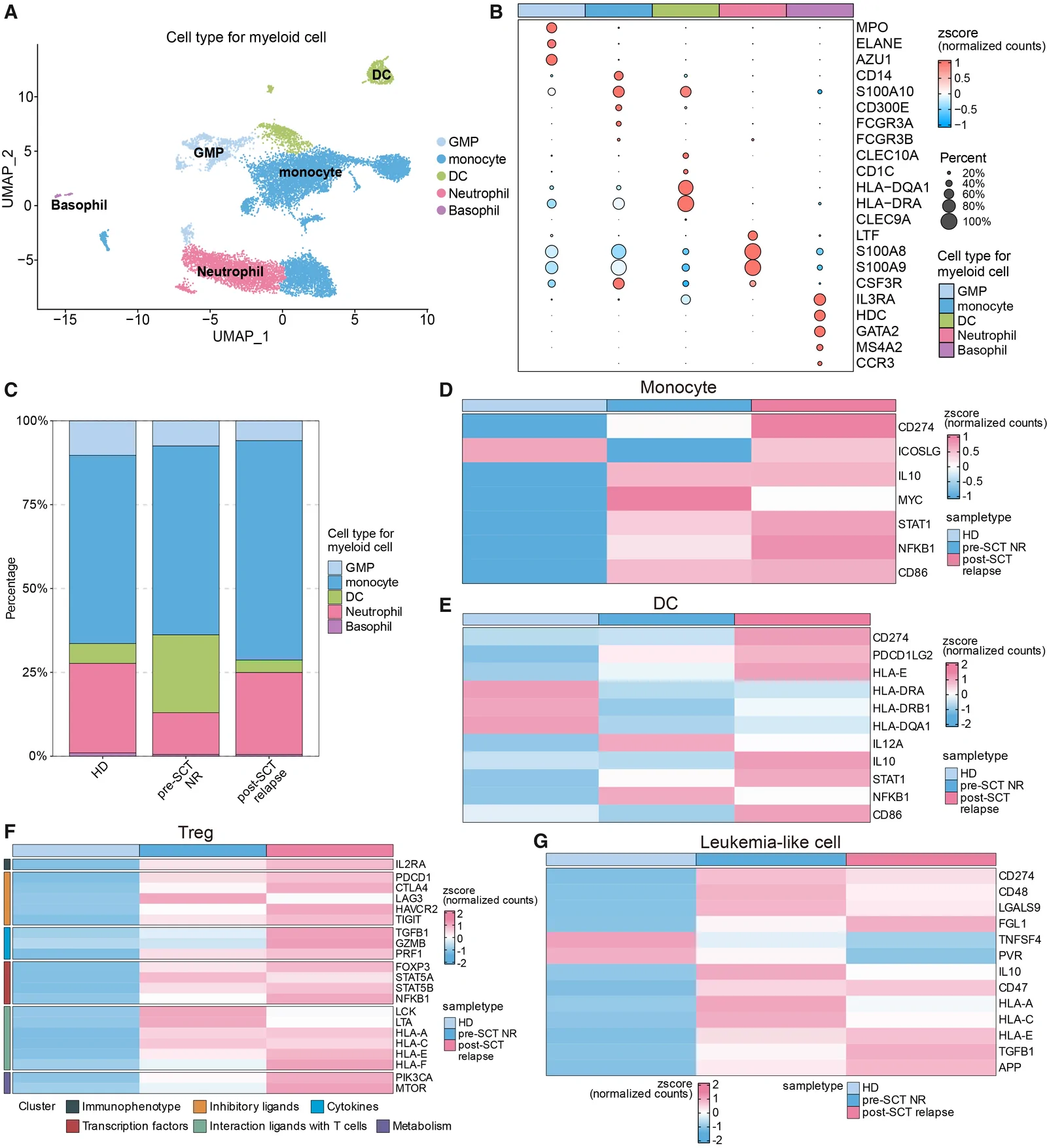
The myeloid cell landscape and immunosuppressive microenvironment of bone marrow in AML patients (A) UMAP plot of all 14,709 myeloid cells colored by myeloid subtypes. (B) Dot plot showing normalized average expression of selected marker genes for different myeloid cell subtypes. The colors represented the average expression levels, and dot sizes represented the percentage expression of selected marker genes in the indicated clusters. (C) Proportion of different myeloid cell subtypes in HD, pre-SCT NR and post-SCT relapse sample types. (D) Heatmap showing normalized average expression of selected characteristic genes related to different functions in monocyte from HD, pre-SCT NR and post-SCT relapse sample types. (E) Heatmap showing normalized average expression of selected characteristic genes related to different functions in DCs from HD, pre-SCT NR and post-SCT relapse sample types. (F) Heatmap showing normalized average expression of selected characteristic genes elated to different functions in Treg from HD, pre-SCT NR and post-SCT relapse sample types. (G) Heatmap showing normalized average expression of selected characteristic genes related to different functions in HSPC from HD sample type, HSC-like blasts from pre-SCT NR and post-SCT relapse sample types.

Analysis of monocytes revealed that, compared with those in HDs, *CD274* and interleukin (IL)-10 were highly expressed, while the ICOS ligand *ICOSLG* was expressed at low levels in groups 1 and 2 (Figure 4D). Compared with those in HDs, MYC, STAT1, and NFKB1 were highly activated and expressed in group 1, whereas only STAT1 and NFKB1 were highly activated and expressed in group 2 (Figures 4D and S6B). In human hepatocellular carcinoma peripheral monocytes, PD-L1 expression is associated with JAK2/STAT1 and JAK2/STAT3/c-MYC activation.^37^ In addition, direct intervention with TFs such as NF-κB might be a promising strategy to reverse the activity of immunosuppressive myeloid components in the tumor microenvironment of AML patients.^38^ The results of our gene regulatory network analysis of MYC, STAT1, and NFKB1 in monocytes suggested that these genes could regulate the expression of ligands of IRs, immunosuppressive cytokines, and ligands involved in the interaction between monocytes and T_ex_ (Figure S6C).

We found that, compared with those in HDs, the DCs in groups 1 and 2 expressed high levels of *CD274*, *PDCD1LG2*, IL-10, and HLA-E but low levels of HLA class II molecules, such as HLA-DRA, HLA-DRB1, and HLA-DQA1 (Figure 4E). Compared with those in HDs, STAT1 and NFKB1 in DCs were both highly activated and expressed in groups 1 and 2 (Figures 4E and S6D). Previous studies have shown that DC-intrinsic IFN-γ-JAK1-STAT1 signaling induces PD-L1 expression, which is required for DCs to convert CD4^+^ T cells into Tregs.^39^ In addition, the DC-intrinsic expression of NF-κB1 governs the ability of DCs to direct Th2 cell differentiation.^40^ Our results suggested that both of the TFs (STAT1 and NFKB1) could regulate the expression of ligands of IRs, inhibitory cytokines of DCs, and ligands that interact between DCs and T_ex_ in groups 1 and 2 (Figure S6E).

In the BM microenvironment, Tregs exert regulatory functions by expressing Foxp3 (via pathways such as the NF-κB pathway) and specific molecules such as PD-1 and CTLA-4. In addition, Tregs bind strongly to IL-2 through CD25 (IL-2Rα), enhancing Treg immune tolerance via the CD25/STAT5 signaling pathway.^41^^,^^42^^,^^43^ Previous studies have suggested that intratumoral infiltrating Tregs promote an exhausted intratumoral T cell state and that the loss of infiltrating Tregs in the tumor microenvironment potentiates the antitumor CD8^+^ T cell effector response and prevents T cell functional exhaustion.^44^^,^^45^ Our results with Tregs demonstrated that compared with those of HDs, IRs such as *PDCD1*, *CTLA4*, *LAG3*, *HAVCR2*, *TIGIT*, inhibitory cytokines (*TGFB1*), granzyme B, and perforin (these two molecules can trigger apoptosis in CD8^+^ T cells and NK cells^46^), *IL2Rα*, key TFs such as *FOXP3*, *STAT5*, and *NFKB1*, and ligands such as LCK and HLA class Ⅰ molecules, which are involved in the interaction between Tregs and T_ex_, were highly expressed in groups 1 and 2; most of these molecules were more prominently expressed in group 2 (Figure 4F).

Furthermore, compared with those in hematopoietic stem and progenitor cells from HDs, the expressions of *CD274*, *CD48*, *FGL1*, *IL-10*, *CD47*, and ligands that interact with T_ex_, such as *TGFB1*, *LGALS9*, and HLA class Ⅰ molecules, in HSC-like blasts were greater, while the expression of the costimulatory molecule ligand *TNFSF4* was lower in HSC-like blasts from groups 1 and 2 (Figure 4G).

In summary, these findings suggest that monocytes, DCs, Tregs, and HSC-like blasts in the BM of R/R AML patients pre- and post-SCT have immunosuppressive effects and constitute an immunosuppressive BM microenvironment.

### Leukemia-like cell-mediated cell-cell interactions induce CD8^+^ T cell exhaustion before and after transplantation

We found that in both groups 1 and 2, leukemia-like cells could directly interact with CD8^+^ Teff and CD8^+^ T_ex_ and exhibited the strongest interaction intensity among all T cell subtypes (Figures 5A and 5B). In group 1, both CellChat and scMLnet analyses revealed that ligand-receptor pairs, such as HLA-E:CD8A/B, MIF:(CD74^+^CD44), LGALS9:PTPRC, and TGFB1:TGFBR1/2, were enriched in the interactions between leukemia-like cells and CD8^+^ T_ex_ (Figure 5C). ScMLnet analysis also predicted that these ligand-receptor pairs might further regulate the activation of NFAT family TFs, which were related to triggering exhaustion in CD8^+^ Teff (Figure 5D). The same analyses in group 2 yielded similar results, except that the TGFB1:TGFBR1/2 ligand-receptor pairs were not detected (Figures 5E and 5F). Our previous results suggested a transition potential of CD8^+^ T_ex_ from CD8^+^ Teff, which prompted us to conduct further analysis of the interactions between leukemia-like cells and CD8^+^ Teff. CellChat and scMLnet analyses predicted that leukemia-like cells interacted with CD8^+^ Teff in both groups 1 and 2 through the same pathways identified in their interactions with the CD8^+^ T_ex_ of the corresponding sample types, thus regulating the expressions of NFAT family TFs in CD8^+^ Teff (Figures 5C, 5E, S7A, and S7B). These results suggest that leukemia-like cells may contribute to exhaustion-associated transcriptional programs in both self-derived and donor-derived CD8^+^ T_ex_ via ligands, such as MIF, LGALS9, and HLA class Ⅰ molecules.

**Figure 5:**
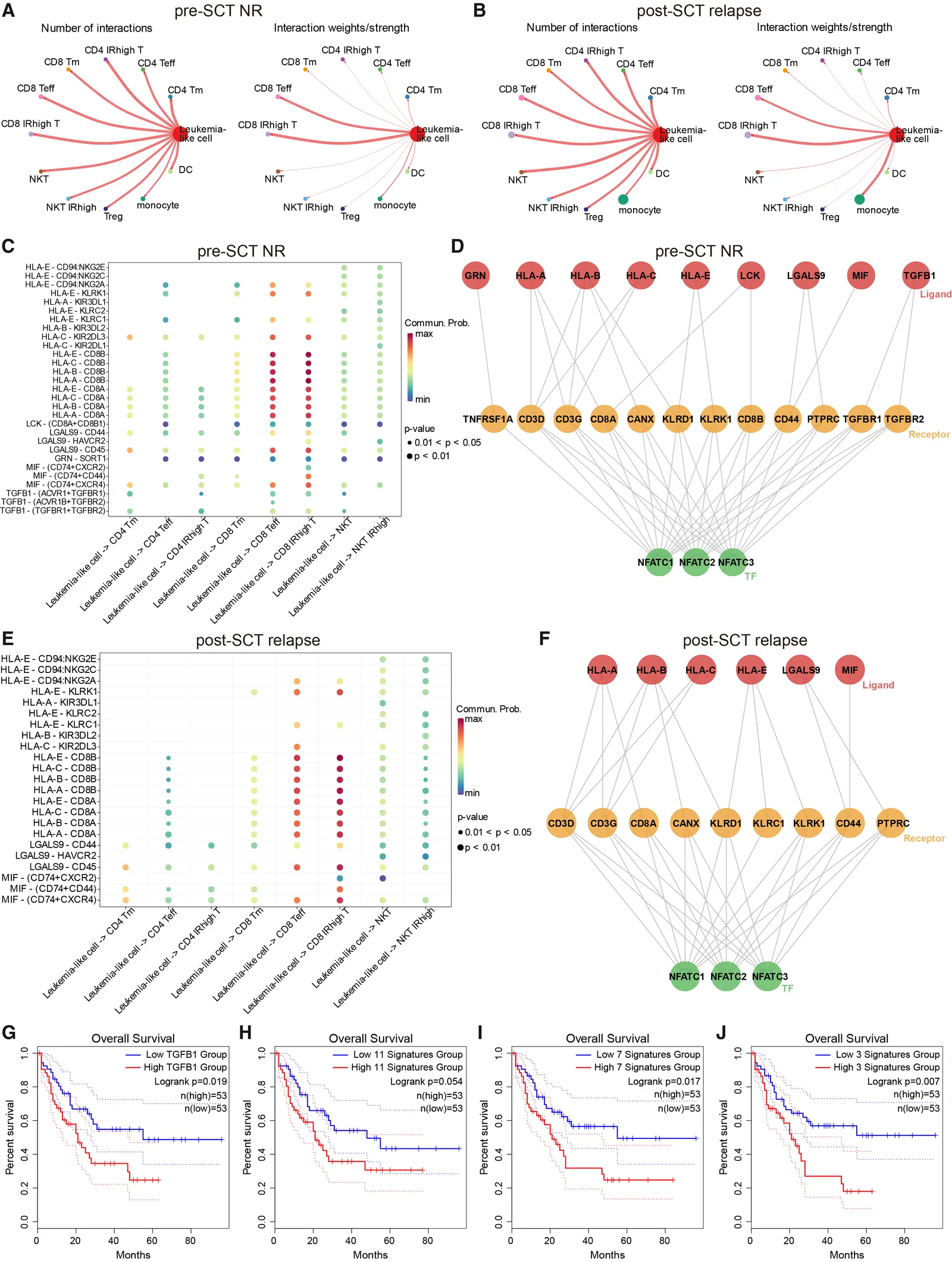
Comparisons of interactions between leukemia-like cells and T cells in AML patients across various disease stages (A) The number of interaction (left) and the weight of interaction (right) of leukemia-like cells in pre-SCT NR patients. (B) The number of interaction (left) and the weight of interaction (right) of leukemia-like cells in post-SCT relapse patients. (C) Dot plot showing the intersections of significant functional ligand-receptor pairs that contribute to the signaling sending from leukemia-like cell to eight T cell subtypes in pre-SCT NR patients. (D) The molecular regulatory network, containing three layers of ligand, receptor, and TF, between leukemia-like cells and CD8^+^ T_ex_ in pre-SCT NR patients. (E) Dot plot showing the intersections of significant functional ligand-receptor pairs that contribute to the signaling sending from leukemia-like cell to eight T cell subtypes in post-SCT relapse patients. (F) The molecular regulatory network, containing three layers of ligand, receptor, and TF, between leukemia-like cells and CD8^+^ T_ex_ in post-SCT relapse patients. (G) Kaplan-Meier analysis of overall survival (OS) of TCGA AML patients. All patients were categorized into two groups based on the median expression level of *TGFB1.* (H) Kaplan-Meier analysis of OS of TCGA AML patients. All patients were categorized into two groups based on the median expression levels of eleven ligands including *MIF*, *LGALS9*, *TGFB1*, *HLA-A*, *HLA-B*, *HLA-C*, *HLA-E*, *LCK*, *GRN*, *CD86*, and *TNFSF13.* (I) Kaplan-Meier analysis of OS of TCGA AML patients. All patients were categorized into two groups based on the median expression levels of seven ligands including *MIF*, *LGALS9*, *TGFB1*, *HLA-A*, *HLA-B*, *HLA-C*, and *HLA-E.* (J) Kaplan-Meier analysis of OS of TCGA AML patients. All patients were categorized into two groups based on the median expression levels of three ligands including *MIF*, *LGALS9*, and *TGFB1.* For circle plots (A and B), different colors represent different cell groups, and the thickness of the lines represents the number of interactions in count plots or the weights/strength of interactions in weight plots between the two cell types. For dot plots (C and E), dot color represents communication probabilities, dot size represents one-sided permutation test *p* values, and empty space indicates that the communication probability is zero. For molecular regulatory network plots (D and F), the ligands were the intersection between CellChat and scMLnet analysis.

We observed that in both groups 1 and 2, leukemia-like cells could also interact with CD8^+^ T_ex_ indirectly; specifically, leukemia-like cells interacted with Tregs, monocytes, and DCs through various ligand-receptor pairs, and these three types of immune cells in turn interacted with CD8^+^ T_ex_ (Figures S7C and S7D). In detail, in group 1, scMLnet analysis predicted that leukemia-like cells may influence the activities of immunosuppressive TF-associated programs in Tregs, including *NFKB1A* and *STAT5A*, which were further associated with *LCK* expression through multiple ligand-receptor pairs, such as MIF:(CD74^+^CD44) and LGALS9:PTPRC (Figures S7E and S7F). LCK-associated signaling interactions between Tregs and CD8^+^ T_ex_ were further predicted to be associated with NFAT family-related transcriptional programs in CD8^+^ T_ex_ (Figures S7G and S7H).

Similarly, in group 1, leukemia-like cells were predicted to modulate the activities of immunosuppressive TF-associated programs in monocytes, such as *MYC*, *NFKB1*, and *STAT1*, which were further associated with the expressions of *CD86*, *TNFSF13*, and *GRN* through multiple ligand-receptor pairs, such as MIF:(CD74^+^CD44), LGALS9:PTPRC, and TGFB1:TGFBR1/2 (Figures S8A and S8B). *CD86*, *TNFSF13*, and *GRN* were further predicted to participate in monocytes-CD8^+^ T_ex_ signaling interactions associated with NFAT family-related transcriptional activity in CD8^+^ T_ex_ (Figures S8C and S8D).

Likewise, in group 1, interactions between leukemia-like cells and DCs involving multiple ligand-receptor pairs, such as MIF:(CD74^+^CD44), LGALS9:PTPRC, and TGFB1:TGFBR1/2, were associated with *NFKB1*- and *STAT1*-related transcriptional programs and the expressions of *CD86* and *GRN* in DCs (Figures S8E and S8F). *CD86* and *GRN* were further predicted to contribute to DC-CD8^+^ T_ex_ communication pathways associated with NFAT-related transcriptional programs in CD8^+^ T_ex_ (Figures S8G and S8H).

Similar interaction patterns were also observed in group 2, where leukemia-like cells were predicted to modulate the activities of corresponding TF-associated programs and the expressions of downstream target genes of Tregs, monocytes and DCs (*LCK* of Tregs; *CD86*, *TNFSF13*, and *GRN* of monocytes; *CD86* and *GRN* of DCs) primarily through *MIF*-, *LGALS9*-, and *TGFB1*-associated signaling pathways. These target genes, in turn, were predicted to interact with CD8^+^ T_ex_ associated with NFAT family-related transcriptional activity in CD8^+^ T_ex_ (Figures S9 and S10). Collectively, these findings indicate that leukemia-like cells may indirectly influence exhaustion-associated transcriptional programs in both self-derived and donor-derived CD8^+^ T cells by modulating intermediary immune cell populations, including Tregs, monocytes, and DCs, which subsequently participate in ligand-receptor-mediated communication with CD8^+^ T_ex_.

Survival analysis was subsequently performed using The Cancer Genome Atlas (TCGA) data from the GEPIA2 for each of the eleven major ligands that induced CD8^+^ T_ex_ pre- and post-SCT. We divided AML patients into two groups on the basis of the median expression of each ligand and observed that only TGFB1 expression was related to survival prognosis (Figure 5G). Next, we combined the ligands into gene sets for survival analysis and found that the gene set containing only the ligands associated with leukemia-like cells was of prognostic significance for survival and was better than TGFB1 alone (Figures 5H and 5I). Moreover, regardless of HLA class Ⅰ molecules, the gene set containing only three ligands (*MIF*, *LGALS9*, and *TGFB1*) had the best prognostic value for AML patients (Figure 5J). In conclusion, our findings indicate that AML patients with high expression of a gene set consisting of MIF, LGALS9, and TGFB1 have poor prognosis.

### Comparison of the effects of different subtypes of leukemia-like cells on the induction of CD8^+^ T_ex_ before and after transplantation

We further analyzed the interactions between different subtypes of leukemia-like cells and other types of immune cells pre- and post-SCT using CellChat. In group 1, compared with other leukemia-like cell subtypes, progenitor-like, GMP-like, DC-like, and HSC-like leukemia cells had stronger interactions with CD8^+^ Teff, CD8^+^ T_ex_, Tregs, monocytes, and DCs (Figures 6A and 6B). On the basis of the interactions between whole leukemia-like cells and other types of immune cells, the HSC-like blasts had the most comprehensive ligand-receptor pairs interacting with CD8^+^ T_ex_, Tregs, monocytes, and DCs, which were the closest to the whole leukemia-like cells; furthermore, the communication probabilities of the interactions associated with HSC-like leukemia cells were relatively high (Figures 6C and 6D). Analysis of group 2 revealed similar results, and the strength of interactions between HSC-like or progenitor-like leukemia cells and other types of immune cells was further highlighted (Figures 6E–6H). These results indicate that the HSC-like leukemia cell subtype plays a more critical role in the interactions between leukemia-like cells and other types of immune cells both before and after transplantation.

**Figure 6:**
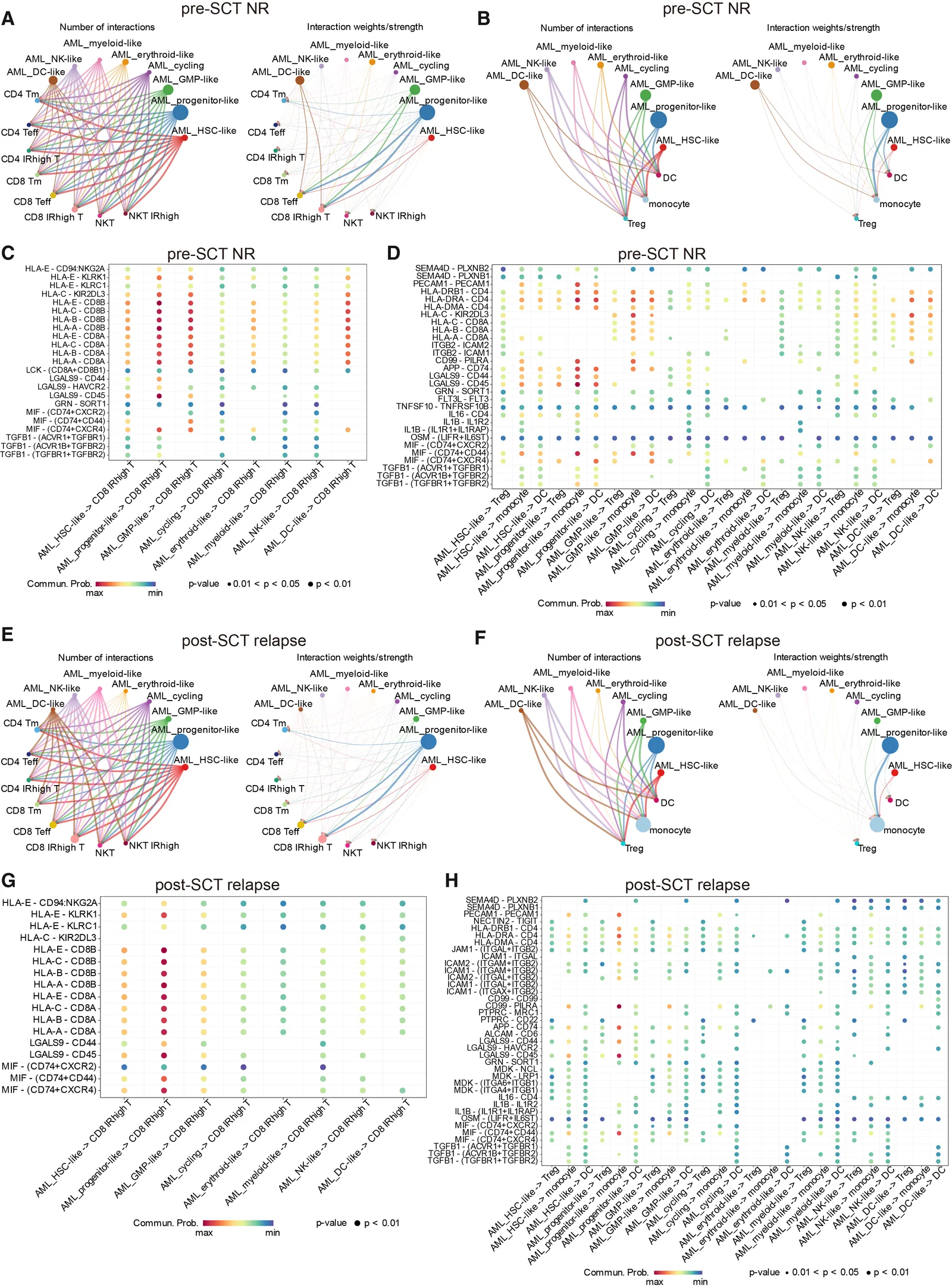
Comparisons of interactions between eight leukemia-like cell subtypes and immune cells in AML patients across various disease stages (A) The number of interaction (left) and the weight of interaction (right) from eight leukemia-like cell subtypes to eight T cell subtypes in pre-SCT NR patients. (B) The number of interaction (left) and the weight of interaction (right) from eight leukemia-like cell subtypes to Treg, monocyte, and DC in pre-SCT NR patients. (C) Dot plot showing the intersections of significant functional ligand-receptor pairs that contribute to the signaling sending from eight leukemia-like cell subtypes to CD8^+^ T_ex_ in pre-SCT NR patients. (D) Dot plot showing the intersections of significant functional ligand-receptor pairs that contribute to the signaling sending from eight leukemia-like cell subtypes to Treg, monocyte, and DC in pre-SCT NR patients. (E) The number of interaction (left) and the weight of interaction (right) from eight leukemia-like cell subtypes to eight T cell subtypes in post-SCT relapse patients. (F) The number of interaction (left) and the weight of interaction (right) from eight leukemia-like cell subtypes to Treg, monocyte, and DC in post-SCT relapse patients. (G) Dot plot showing the intersections of significant functional ligand-receptor pairs that contribute to the signaling sending from eight leukemia-like cell subtypes to CD8^+^ T_ex_ in post-SCT relapse patients. (H) Dot plot showing the intersections of significant functional ligand-receptor pairs that contribute to the signaling sending from eight leukemia-like cell subtypes to Treg, monocyte, and DC in post-SCT relapse patients. For circle plots (A, B, E, and F), different colors represent different cell groups, and the thickness of the lines represents the number of interactions in count plots or the weights/strength of interactions in weight plots between the two cell types. For dot plots (C, D, G, and H), dot color represents communication probabilities, dot size represents one-sided permutation test *p* values, and empty space means that the communication probability is zero.

In summary, leukemia-like cells were predicted to interact with self-derived CD8^+^ T cells through both direct pathways (mainly involving ligands such as MIF, LGALS9, and HLA class Ⅰ molecules) and indirect pathways (mainly involving ligands such as MIF, LGALS9, and TGFB1), which were associated with increased activities of NFATC1- and NFATC3-related transcriptional programs together with elevated TOX and EOMES expression in CD8^+^ T_ex_ (Figure 7A and Data S5). Likewise, leukemia-like cells were predicted to interact with donor-derived CD8^+^ T cells through similar ligand-receptor pathways, which were associated with enrichment of NFATC2-related regulatory signatures and increased NR4A2 expression in donor-derived CD8^+^ T_ex_ (Figure 7B and Data S6). Among all leukemia-like cell subsets, HSC-like cells were predominant in interactions with CD8^+^ T_ex_, Tregs, monocytes, and DCs.

**Figure 7:**
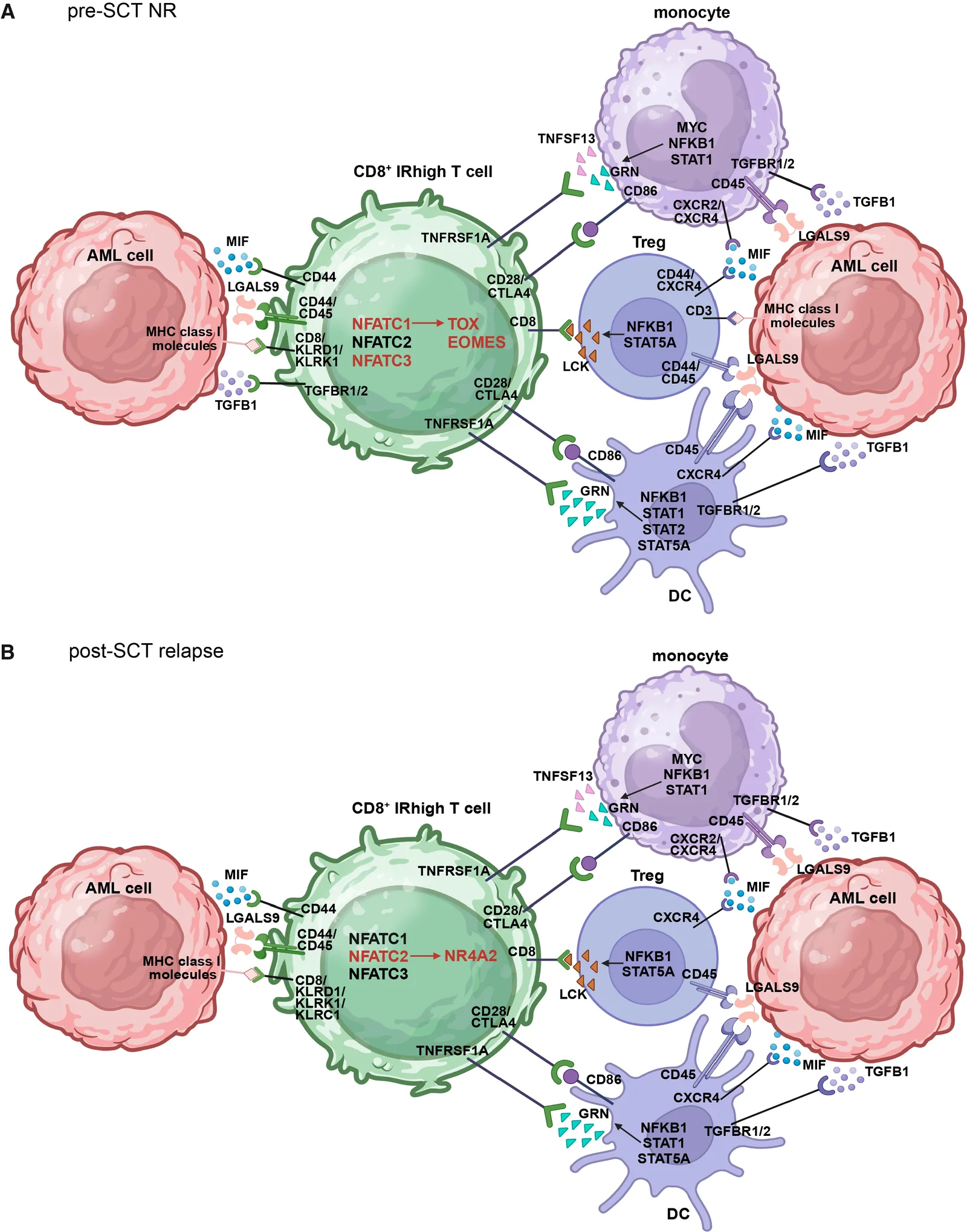
Summary of multicellular interaction network pre- and post-transplantation (A) Summary of multicellular interaction network depicting regulation of CD8^+^ T_ex_ by other types of cells in pre-SCT NR patients. In detail, self-derived CD8^+^ T_ex_ were characterized by enrichment of NFATC1/NFATC3-associated transcriptional programs together with increased TOX and EOMES expression. Computational cell-cell interaction analyses predicted that leukemia-like cells may directly interact with CD8^+^ T_ex_ through ligand-receptor pairs, such as HLA-E:CD8A/B, MIF:(CD74^+^CD44), LGALS9:CD45, and TGFB1:TGFBR1/2, which were associated with increased activity of NFAT family-related transcriptional programs in CD8^+^ T_ex_. In addition, leukemia-like cells were predicted to indirectly interact with CD8^+^ T_ex_ via Tregs, monocytes, and DCs through ligand-receptor pairs including MIF:(CD74^+^CD44), MIF:(CD74+CXCR4), LGALS9:CD45, and TGFB1:TGFBR1/2. Genes enriched in these intermediate immune cell populations (including *LCK* in Tregs; *CD86*, *TNFSF13*, and *GRN* in monocytes; *CD86* and *GRN* in DCs) may further participate in signaling interactions associated with NFAT-related transcriptional activity in CD8^+^ T_ex_. (B) Summary of multicellular interaction network depicting regulation of CD8^+^ T_ex_ by other types of cells in post-SCT relapse patients. In detail, donor-derived CD8^+^ T_ex_ were characterized by enrichment of NFATC2-associated transcriptional programs together with elevated NR4A2 expression. Computational cell-cell interaction analyses predicted that leukemia-like cells may directly interact with CD8^+^ T_ex_ through ligand-receptor pairs, such as HLA-E:CD8A/B, MIF:(CD74^+^CD44), and LGALS9:CD45, which were associated with increased activity of NFAT family-related transcriptional programs in CD8^+^ T_ex_. Besides, leukemia-like cells were predicted to indirectly interact with CD8^+^ T_ex_ via Tregs, monocytes, and DCs through ligand-receptor pairs including MIF:(CD74^+^CD44), MIF:(CD74+CXCR4), LGALS9:CD45, and TGFB1:TGFBR1/2. Genes enriched in these intermediate immune cell populations (including *LCK* in Tregs; *CD86*, *TNFSF13*, and *GRN* in monocytes; *CD86* and *GRN* in DCs) may further participate in signaling interactions associated with NFAT-related transcriptional activity in CD8^+^ T_ex_.

## Discussion

In this study, we compared CD8^+^ T_ex_ in the immunosuppressive BM microenvironment between AML patients with pre-transplant NR and those with post-transplant relapse. We found that self-derived CD8^+^ T_ex_ were characterized by enrichment of NFATC1/NFATC3-associated transcriptional programs together with increased TOX and EOMES expression, whereas donor-derived CD8^+^ T_ex_ showed enrichment of NFATC2-associated signatures and elevated NR4A2 expression. Moreover, AML cells interacted with both types of CD8^+^ T_ex_ through similar signaling pathways involving MIF, LGALS9, and TGFB1. Our work provides a comparative characterization of distinct transcriptional programs associated with self-versus donor-derived CD8^+^ T_ex_ and highlights potential targets for future studies aimed at modulating T cell exhaustion.

Available data indicate that prolonged antigen exposure induces T cell exhaustion through TFs such as TOX, NR4A, and EOMES.^5^^,^^24^^,^^27^ In AML, self-derived CD8^+^ T_ex_ were characterized by enrichment of NFATC1/NFTC3-TOX/EOMES-associated transcriptional programs, whereas donor-derived CD8^+^ T_ex_ exhibited distinct NFATC2-NR4A2-related regulatory signatures. Studies have shown that loss of TOX or NR4A enhances cytokine production and reduces IRs in T cells.^27^^,^^47^ Moreover, PD-1/PD-L1 blockade acts mainly through cytosolic signaling rather than reshaping TF-related chromatin accessibility in CD8^+^ T_ex_.^48^ Overall, the aforementioned findings reported by others and us may have implications for understanding unsatisfactory responses to immune checkpoint inhibitors (ICIs) in patients with AML^6^^,^^49^ and suggest that targeting TOX in self-derived CD8^+^ T_ex_ and NR4A2 in donor-derived CD8^+^ T_ex_ could be a potential strategy to reverse exhaustion.

In this study, we revealed that leukemia cells interact with both self- and donor-derived CD8^+^ T_ex_—directly or indirectly via monocytes, DCs, and Tregs—primarily through MIF, LGALS9, and TGFB1. Notably, direct TGF-β1-mediated interactions were absent between leukemia cells and donor-derived CD8^+^ T_ex_ after transplantation. Previous studies have also demonstrated the role of MIF, LGALS9, and TGFB1 in inducing T cell exhaustion.^19^^,^^21^^,^^22^ In diffuse large B cell lymphoma, the interactions between macrophages and T_ex_ (mediated by LGALS9:HAVCR2, CD86:CTLA4, and NECTIN2:TIGIT) were enhanced during disease progression.^19^ In melanoma models, increased MIF expression in tumors resulted in the recruitment of CD74^+^ macrophages, leading to a pronounced T cell exhaustion phenotype and consequently attenuating antitumor immunity; moreover, Mif-knockout tumor cells in mice reduced the exhaustion and increased the function of cytotoxic T cells.^22^ In addition, hepatocellular carcinoma cells promoted hepatic stellate cells to secrete TGF-β1, thus facilitating CD4^+^ T cell exhaustion and Treg differentiation.^21^ Notably, the role of TGF-β signaling in T cell exhaustion may be more complex and context dependent than a purely immunosuppressive effect. Previous studies have shown that TGF-β signaling can contribute not only to exhaustion-associated programs but also to the maintenance of stem-like precursor T_ex_ state.^50^^,^^51^ Therefore, although our data identified TGF-β1-associated signaling interactions in AML, the precise functional impact of TGF-β signaling on self- and donor-derived CD8^+^ T_ex_ populations requires further investigation. Overall, our findings suggest that MIF-, LGALS9-, and TGF-β1-associated signaling pathways may contribute to the establishment of exhaustion-related states in CD8^+^ T cells in AML, yet the specific ligand-receptor pairs differ by disease contexts. This diversity in functional interactions beyond conventional immune checkpoints, such as PD-1/PD-L1, may help explain the limited efficacy of currently available ICIs in AML^6^^,^^49^ and may highlight MIF, LGALS9, and TGF-β1 as potential therapeutic targets.

Using the scMLnet package developed by Cheng et al.,^52^ we were able to link ligand-receptor interactions with their downstream TFs in CD8^+^ T_ex_. We found that in direct pathways, AML cells were predicted to interact with CD8^+^ T_ex_ via ligands involving MIF, LGALS9, and HLA class Ⅰ molecules, which were associated with NFAT family-related transcriptional programs in CD8^+^ T_ex_. In indirect pathways, intermediary immune cell populations, including Tregs, monocytes, and DCs, were predicted to communicate with CD8^+^ T_ex_ through ligands such as LCK and CD86, thereby potentially contributing to exhaustion-associated transcriptional states in CD8^+^ T_ex_. As described in previous studies, CD8^+^ T cell exhaustion is driven by TCR signaling that leads to NFAT-TCF1 activity followed by activation of the TOX/NR4A axis.^53^^,^^54^

In summary, we have described two distinct circumstances of AML immune environments with respect to cell composition and phenotypes, T cell function, transcriptional regulatory profiles, and cellular crosstalk. Our results suggest that the activities of NFAT-, TOX-, EOMES-, and NR4A2-related transcriptional programs can help to distinguish self-derived and donor-derived CD8^+^ T_ex_ and facilitate targeted interventions for exhaustion reversion. In addition, targeting key intercellular interaction molecules (e.g., MIF, galectin-9, TGF-β1, TOX, and NR4A2) might represent a strategy for reversing T cell exhaustion.

### Limitations of the study

Our study has several limitations. First, the findings of this study were primarily inferred computationally, and the functional involvement of these identified regulators (e.g., TFs and ligands) in T cell exhaustion in AML will require further experimental validation. Second, expanding the sample size will increase statistical power and reduce the likelihood of observing spurious relationships between variables, particularly for relatively rare cell types. A larger cohort would also allow for better balancing of different AML subtypes and risk stratification among patients, thus strengthening the generalizability of our results.

## Resource availability

### Lead contact

Requests for further information, resources, and reagents should be directed to and will be fulfilled by the lead contact, Ying-Jun Chang (rmcyj@bjmu.edu.cn).

### Materials availability

This study did not generate new unique reagents.

### Data and code availability

• The scRNA-seq and scTCR-seq data generated during this study have been deposited in the Genome Sequence Archive (GSA), National Genomics Data Center (NGDC), China National Center for Bioinformation (CNCB) under the accession number HRA016119.
• This paper does not report original code.
• Any additional information required to reanalyze the data reported in this paper is available from the lead contact upon request.

## Acknowledgments

This study was supported by grants from the 10.13039/501100001809National Natural Science Foundation of China (82293630, 82293633, and 62273364); the 10.13039/501100009592Beijing Municipal Science and Technology Commission (Z221100007422008). We would like to thank the American Journal Experts (www.aje.com) for assistance in editing this manuscript. Graphic abstract, Figures 1A and 7 were created with BioRender.com.

## Author contributions

Y.-J.C. and X.-J.H. designed the study. Y.-J.C., C.-Z.Y., H.C., Y.-H.C., F.-R.W., Y.-F.C., Y.-Q.S., Y.-Y.Z., and M.L. collected the data and performed single-cell sequencing. C.-Z.Y. and Y.-L.C. performed the data analysis. X.-H.Z., L.-P.X., X.-D.M., K.-Y.L., X.-Q.S., and X.-Y.Z. supervised the data analysis. Y.-J.C., Y.W., and X.-S.Z. drafted the manuscript. All authors contributed to data interpretation, manuscript preparation, and approval of the final version.

## Declaration of interests

The authors declare no competing interests.

## STAR★Methods

### Key resources table

**Table:** 

REAGENT or RESOURCE	SOURCE	IDENTIFIER
**Biologicalsamples**		

Individuals with AML and healthy donors’ bone marrow samples	Peking University People’s Hospital	N/A

**Criticalcommercialassays**		

Ficoll solution	TBD	Cat #LTS1077
Chromium SingleCell 5′ Library and Gel Bead Kit	10× Genomics	Cat #PN-1000263

**Depositeddata**		

Data files for single-cell RNA sequencing (raw data)	This paper	https://ngdc.cncb.ac.cn/search/specific?db=hra&q=HRA016119
Data files for single-cell TCR sequencing (raw data)	This paper	https://ngdc.cncb.ac.cn/search/specific?db=hra&q=HRA016119

**Softwareandalgorithms**		

Cell Ranger (v7.1.0)	10× Genomics	https://www.10xgenomics.com/support/software/cell-ranger/
R (v4.3.0)	CRAN	https://www.r-project.org/
Seurat (v4.3.0)	Satija Lab	https://satijalab.org/seurat/
harmony (v1.0.2)	Korsunsky et al.^55^	https://github.com/immunogenomics/harmony
SingleR	Aran et al.^56^	https://github.com/SingleR-inc/SingleR
copykat (v1.1.0)	Gao et al.^57^	https://github.com/navinlabcode/copykat
CytoTRACE (v0.3)	Gulati et al.^58^	https://github.com/gunsagargulati/CytoTRACE
clusterProfiler (v4.8.1)	Yu et al.^59^	https://github.com/YuLab-SMU/clusterProfiler
monocle3 (v1.3.1)	Cao et al.^60^	https://github.com/cole-trapnell-lab/monocle3
Slingshot (SCP v0.4.8)	Street et al.^35^	https://github.com/zhanghao-njmu/SCP
SCENIC (v0.12.1)	Aibar et al.^61^	https://github.com/aertslab/pySCENIC
iGraph (v1.4.2)	Yan et al.^62^	https://github.com/igraph/igraph
CellChat (v1.6.1)	Jin et al.^63^	https://github.com/sqjin/CellChat
scMLnet (v3.0)	Cheng et al.^52^	https://github.com/SunXQlab/scMLnet3.0/
scRepertoire (v1.10.1)	Borcherding et al.^64^	https://github.com/BorchLab/scRepertoire
Startrac (v0.1.0)	Zhang et al.^26^	https://github.com/Japrin/STARTRAC

### Experimental model and study participant details

#### Human specimens

For the scRNA-seq and scTCR-seq studies, twelve bone marrow samples (matched pre-SCT NR and post-SCT relapse samples from four AML patients, including two males and two females with a median age of 45.5 years (range, 25–58), as well as samples from four healthy donors (HDs), including two males and two females with a median age of 34.5 years (range, 32–43)) were obtained from Peking University People’s Hospital. The available clinical characteristics of the four AML patients are summarized in Figure S1A and Data S1. All participating patients and HDs provided signed informed consent. The study was performed in accordance with the Declaration of Helsinki and was approved by the Institutional Review Board of Peking University (Approval No. 2023PHB132-001).

### Method details

#### Preparation of single-cell suspensions

Ficoll solution (TBD) and density gradient centrifugation were used to isolate bone marrow mononuclear cells (BMMCs) from the iliac crest aspirates of AML patients and HDs according to the manufacturer’s instructions. Briefly, 4 mL of fresh bone marrow blood was collected in an EDTA anticoagulant tube and subsequently layered onto Ficoll. After centrifugation, the BMMCs remaining at the plasma-Ficoll interface were transferred to a new tube with caution and washed twice with DPBS before being sent for further processing.

#### Library preparation and sequencing

Single-cell RNA-seq and TCR-seq libraries were prepared using the Chromium SingleCell 5′ Library and Gel Bead Kit according to the manufacturer’s instructions. Briefly, the single-cell suspension was modified to a concentration of 700–1200 cells/μL (viability ≥85%) as determined by a Countess Ⅱ Automated Cell Counter (Thermo Fisher Scientific, USA). Cells were captured in droplets during targeted cell recovery. After the reverse transcription step, the emulsions were broken, and the resulting barcoded cDNA was subsequently purified with Dynabeads, followed by polymerase chain reaction (PCR) amplification. Amplified cDNA was subsequently used for both 5′ gene expression library construction and TCR enrichment. For gene expression library construction, 50 ng of amplified cDNA was fragmented and end-repaired, double-sized, selected with SPRIselect beads, and sequenced on a NovaSeq platform (Illumia) to generate 150 bp paired-end reads. For TCR library construction, TCR transcripts were amplified with 10 cycles of PCR using the DNA primers provided in the kit. VDJ region-enriched libraries were size selected with SPRI beads and sequenced on an Illumina NovaSeq 6000 instrument using 150 paired-end reads.

#### Data preprocessing and quality control

The raw reads were analyzed using Cell Ranger v7.1.0 and aligned to the GRCh38 human reference genome sequence. Gene expression and TCR profiles were quantified using the “count” and “vdj” subcommands of the Cell Ranger software package with default parameters.

The above process produced raw unique molecular identifier (UMI) count matrices, and twelve pieces of 10X-derived data (both pre-SCT NR and post-SCT relapse samples from four AML patients and samples from four HDs) were obtained. Afterward, the resulting gene-by-cell matrices from Cell Ranger were transformed into Seurat objects (Seurat v4.3.0), and downstream analysis was conducted in R v4.3.0 unless otherwise stated. All the Seurat objects were subsequently merged and filtered to exclude cells with UMI numbers <500, expressed genes <250, or with ≥10% of the reads mapped to mitochondrial genes. In addition, to remove potential doublets, we also filtered out single cells with more than 5000 genes detected. In addition, only genes expressed in more than ten cells were retained. Finally, 95,426 cells were retained for downstream analyses.

#### scRNA-seq clustering and cell type annotation

After quality control, UMI counts were log normalized by the NormalizeData function, and the top 2,000 features with high cell-to-cell variation were calculated using the FindVariableFeatures function. Afterward, the ScaleData function was applied to the scale and center features in the datasets, and the RunPCA function was used with default parameters to reduce dimensionality. The R package harmony (version 1.0.2)^55^ was used for batch correction to avoid batch effects across samples. Subsequently, the RunUMAP function was implemented for nonlinear dimensional reduction, and the FindNeighbors and FindClusters functions were used for cluster analysis of the data.

After all cells were projected into two-dimensional spaces by UMAP, with the exception of suspected leukemia-like cells, the normal hematopoietic cells were clustered together according to common features. However, owing to the crossover of gene expression profiles between leukemia-like cells and normal bone marrow cells, the characteristics of leukemia-like cells varied among individuals, and leukemia-like cells did not cluster together and were mixed with normal myeloid lineage cells, erythrocytes, and T cells. Therefore, a preliminary analysis was performed on each single sample from every AML patient in combination with four HDs independently to discriminate leukemia-like cells from their normal hematopoietic counterparts. Cell-type annotation and leukemia-like cell inference were performed through an integrative strategy rather than relying on a single method.^56^^,^^57^^,^^58^^,^^59^^,^^60^^,^^61^^,^^62^^,^^63^^,^^64^ Specifically, we considered the following features: (i) sample-origin distribution within clusters, where putative leukemia-like clusters were composed predominantly of cells derived from AML patient samples with minimal representation from HD samples; (ii) reference-based annotation using SingleR^56^ with the NovershternHematopoieticData^65^ dataset, which showed that putative leukemia-like cells frequently exhibited HSC/progenitor-like transcriptional features; (iii) single-cell copy number variation (CNV) analysis using the R package copykat (version 1.1.0),^57^ with HD-derived cells used as references and default parameters to distinguish putative malignant from nonmalignant populations based on copy-number karyotyping of aneuploid tumors; (iv) expression of established cell type-associated marker genes; and (v) differentiation-state prediction using the R package CytoTRACE (version 0.3)^58^ with default parameters, which was used as a complementary analysis rather than as definitive evidence of leukemic identity, where leukemia-like cells tended to be less differentiated than normal cells were.^66^ Based on the integrated results from these analyses, cells were classified into seven major cell types. An example of the annotation workflow using a pre-SCT NR sample from patient01 combined with four HDs is shown in Figures S1C–S1I. Then, we combined the cell type annotation results from all the samples. Three major cell types, namely, leukemia-like cells, T cells, and myeloid cells, were subsequently subset and further clustered into subclusters to detect heterogeneity within every cell type.

#### Differential gene expression and functional enrichment

Differentially expressed genes (DEGs) of the leukemia-like cell subgroup were recognized by the “FindMarkers” function in Seurat (version 4.3.0) using the Wilcox method. Genes whose |logFC| > 0.25 and adjusted *p* value <0.05 were considered DEGs and are listed in Supplementary Data 3. GO enrichment analysis was performed on these DEGs using the R package clusterProfiler (version 4.8.1)^59^ with default parameters.

#### Pseudotime analysis

Different trajectory inference methods were selected according to the transcriptional characteristics and lineage structures of distinct cell populations.^67^

Pseudotime trajectory analysis was performed for leukemia-like cells using the R package monocle3 (version 1.3.1) to reconstruct developmental-like trajectories across heterogeneous states.^60^ The root was determined by the mean expression levels of CD34, HOPX, and FAM30A, which were recently identified as marker genes of HSC-like leukemia cells.^15^^,^^16^^,^^17^

To study the trajectory of CD8^+^ T_ex_ cell subtypes, pseudotime analysis was performed on this group of cells using the Slingshot^35^ method provided by the “RunSlingshot” function in the R package SCP (version 0.4.8) to infer transitions among relatively discrete functional and exhaustion-associated states. The group variable was set as the subtype of CD8^+^ T_ex_, and the T_ex_^prog^ subtype was set as the starting point according to previous studies.

#### Single-cell gene set expression scores

The expression scores of gene sets, including exhaustion, cytotoxicity, and proliferation, were calculated by the “AddModuleScore” function provided by Seurat with default parameters. The three reference gene sets are shown in Supplementary Data 4.

#### Gene regulatory network and transcription factor (TF) analysis

To measure the difference between cell clusters or sample types on the basis of TFs or their target genes, SCENIC^61^ was performed on designated cells. In this work, the pySCENIC package (version 0.12.1) in Python (version 3.7.16) was used to assess the enrichment of transcription factors and the activity of regulons. The SCENIC workflow consisted of three steps: (i) identifying coexpression modules between the TF and potential target genes, followed by visualizing the gene regulatory network using the function “graph_from_data_frame” in the R package iGraph (version 1.4.2)^62^ and selecting the target genes of interest for display; (ii) inferring direct target genes on the basis of the potential targets for which the motif of the corresponding TF was significantly enriched; and (iii) calculating the regulon activity score in every single cell using the area under the recovery curve. In addition to directly comparing the activity of specific regulons across different cell clusters or sample types, we also adapted an entropy-based strategy to generate a regulon-specific score (RSS) to further quantify the cell-type specificity or the sample-type specificity of a regulon.^32^ The motif rankings for human genes were obtained from the website https://resources.aertslab.org/cistarget/.

#### Cell–cell communication analysis

##### CellChat

To assess cell-to-cell communication, we first utilized the CellChat package (version 1.6.1),^63^ which includes a comprehensive database of curated intercellular signaling interactions, to identify potential ligand–receptor interactions between cells. The inference of cell–cell communication through CellChat includes identifying differentially expressed signaling genes, calculating the probability of intercellular communication, and discovering statistically significant intercellular communications.^32^ We used CellChat to analyze pre-SCT NR and post-SCT relapse samples from AML patients separately.

##### scMLnet

To analyze the downstream TFs and target genes of potential ligand–receptor pairs, we further employed the scMLnet package (version 3.0)^52^ (https://github.com/SunXQlab/scMLnet3.0/) with an updated prior knowledge database^68^ to construct a multilayer network by integrating the intercellular pathways (ligand–receptor interactions) and intracellular subnetworks (receptor–TF pathways and TF–target gene regulations).

In this study, leukemia-like cells, Tregs, monocytes, and DCs were used as the main sender cells, and CD8^+^ effector T cells, CD8^+^ T_ex_, Tregs, monocytes, and DCs were used as the main receiver cells. For cell-to-cell interactions, the ligands shown were derived from the intersection of CellChat and scMLnet analyses. With respect to the receptor-TF pathways in CD8^+^ T cells, we focused on the regulation of NFAT family TFs (NFATC1, NFATC2, and NFATC3).

#### Survival analysis using the public dataset

To test whether the different identified ligand combinations could stratify AML patients with respect to overall survival, we used the AML RNA sequencing dataset from The Cancer Genome Atlas (TCGA). We utilized Gene Expression Profiling Interactive Analysis 2 (GEPIA2, http://gepia2.cancer-pku.cn/)^69^ for survival analysis. Patients with AML were stratified into a high-expression group and a low-expression group on the basis of the median expression of the signatures. Kaplan–Meier analysis was subsequently employed to assess the correlation between expression subgroups and overall survival outcomes.

#### Single-cell TCR clonotype analysis

TCR clonotype analysis was conducted using the R package scRepertoire (version 1.10.1)^64^ on the basis of the TCR-seq libraries. Each unique TRA(s)-TRB(s) pair was defined as a clonotype in the TCR. If one clonotype was present in at least two cells, the cells possessing this clonotype were regarded as clonal.^70^

T cell clonotype data were integrated with the T cell phenotypic data on the basis of the shared cell barcodes. The clonal homeostasis occupied by clonotypes of specific proportions was first identified and visualized using the clonalHomeostasis function. In addition, we applied the clonalOverlay function to visualize the clonal expansion of different sample types. Next, depending on the clonalOverlap function, the clonotype overlap between T cell subtypes was calculated and visualized using overlap index methods. Moreover, we used the alluvialClonotypes function to explore the dynamics among different sample types, T cell subtypes, and clonotypes.

To investigate the transitions between T cell subtypes, we first utilized the getCirclize function to determine the numbers of clonotypes shared across them. Analysis was subsequently performed using the R package Startrac (version 0.1.0)^26^ to assess the cell fate state transition of T cell subtypes upon TCR tracking, which was determined by the “pIndex.tran” index.

### Quantification and statistical analysis

The quantification and statistical analyses in this article were conducted using R software (version 4.3.0). The Wilcoxon rank-sum test was used for comparisons of gene expression levels, gene set expression scores, LSC17 scores, and proportions of cell clusters in different sample types. A *p* value <0.05 was considered to indicate statistical significance.

## Supplemental information

Document S1. Figures S1–S10

Data S1. Summary of sequencing depth and data quality of single-cell RNA datasets

Data S2. Differentially expressed genes across leukemia-like cell clusters

Data S3. Differentially expressed genes between non-HSC-like blasts and HSC-like blasts

Data S4. Reference gene sets for T cell exhaustion, cytotoxicity, and proliferation

Data S5. Inferred ligand-receptor pairs for interactions between different cell types in pre-SCT NR samples

Data S6. Inferred ligand-receptor pairs for interactions between different cell types in post-SCT relapse samples
