# Supplementary material for: Single-cell dissection of self-derived versus donor-derived T cell exhaustion niches in patients with acute myeloid leukemia
Source: iScience. 2026 Oct 16;29(11):117918. doi: 10.1016/j.isci.2026.117918 (PMC13691313; doi:10.1016/j.isci.2026.117918)
Supplement: Supplementary file 1 [file mmc1.pdf]

iScience, Volume *29*

Supplemental information

Single-cell dissection of self-derived versus donor-derived T cell exhaustion niches in patients with acute myeloid leukemia

Chun-Zi Yu, Yu-Lun Chen, Xiao-Hui Zhang, Lan-Ping Xu, Yu Wang, Huan Chen, Yu-Hong Chen, Feng-Rong Wang, Yu-Qian Sun, Yuan-Yuan Zhang, Meng Lv, Xiao-Dong Mo, Yi-Fei Cheng, Kai-Yan Liu, Xiao-Su Zhao, Xiao-Qiang Sun, Xiang-Yu Zhao, Xiao-Jun Huang, and Ying-Jun Chang

Figure S1

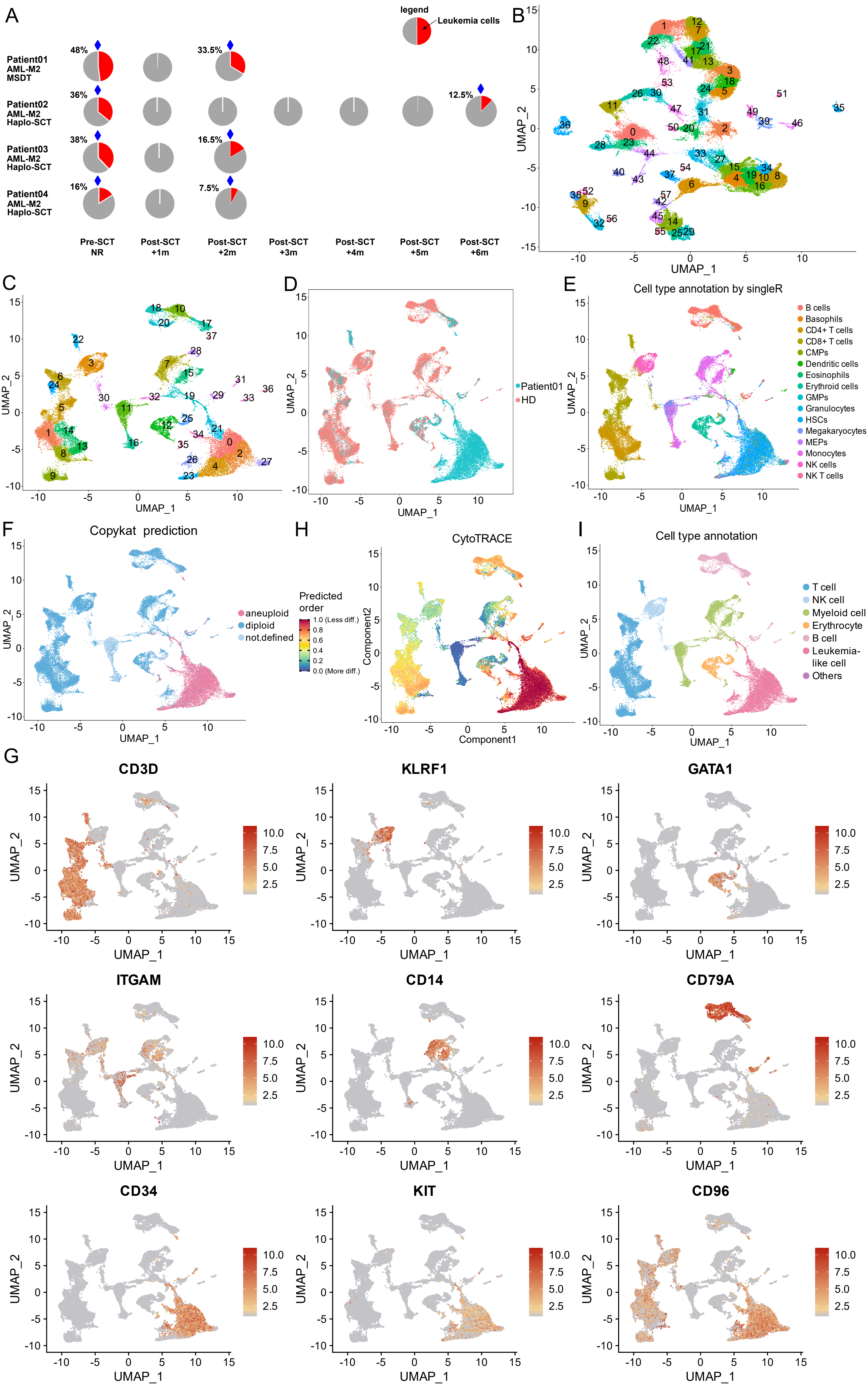
A
legend
Leukemia cells
Patient01 AML-M2 MSDT
Patient02 AML-M2 Haplo-SCT
Patient03 AML-M2 Haplo-SCT
Patient04 AML-M2 Haplo-SCT
48%
33.5%
36%
12.5%
38%
16.5%
16%
7.5%
Pre-SCT NR
Post-SCT +1m
Post-SCT +2m
Post-SCT +3m
Post-SCT +4m
Post-SCT +5m
Post-SCT +6m
B
C
D
Patient01
HD
E
Cell type annotation by singleR
B cells
Basophils
CD4+ T cells
CD8+ T cells
CMPs
Dendritic cells
Eosinophils
Erythroid cells
GMPs
Granulocytes
HSCs
Megakaryocytes
MEPs
Monocytes
NK cells
NK T cells
F
Copykat prediction
aneuploid
diploid
not.defined
H
CytoTRACE
Predicted order
1.0 (Less diff.)
0.0 (More diff.)
Component1
Component2
I
Cell type annotation
T cell
NK cell
Myeloid cell
Erythrocyte
B cell
Leukemia-like cell
Others
G
CD3D
KLRF1
GATA1
ITGAM
CD14
CD79A
CD34
KIT
CD96
UMAP_1
UMAP_2

Figure S1. A single-cell transcriptomic atlas of bone marrow environments in AML patients.

A Table summarizing four AML patients' clinical features and timepoints selected for single-cell RNA sequencing (blue diamonds). AML, acute myeloid leukemia; MSDT, HLA-matched sibling donor transplantation; haplo-SCT, haploidentical stem cell transplantation; MRD, measurable residual disease; SCT, stem cell transplantation; NR, non-remission.

B UMAP plot of scRNA-seq data from all 95,426 cells colored by cluster from initial high-resolution Seurat clustering.

C-I A cell type annotation example of the pre-SCT NR sample from patient01 combined with four HDs.

C UMAP plot of scRNA-seq data colored by cluster from initial high-resolution Seurat clustering.

D UMAP plot of scRNA-seq data colored by sampletype.

E UMAP plot of scRNA-seq data colored by cell type annotation using singleR.

F UMAP plot of scRNA-seq data colored by cell ploidy prediction using Copykat.

G UMAP plot showing transcriptional expression distribution of main cell type markers.

H Predicted order (color gradient) for cells projected on UMAP embedding.

I UMAP plot of scRNA-seq data colored by cell type. Cell types were annotated based on the combination of results from **E** to **H**.

Figure S2

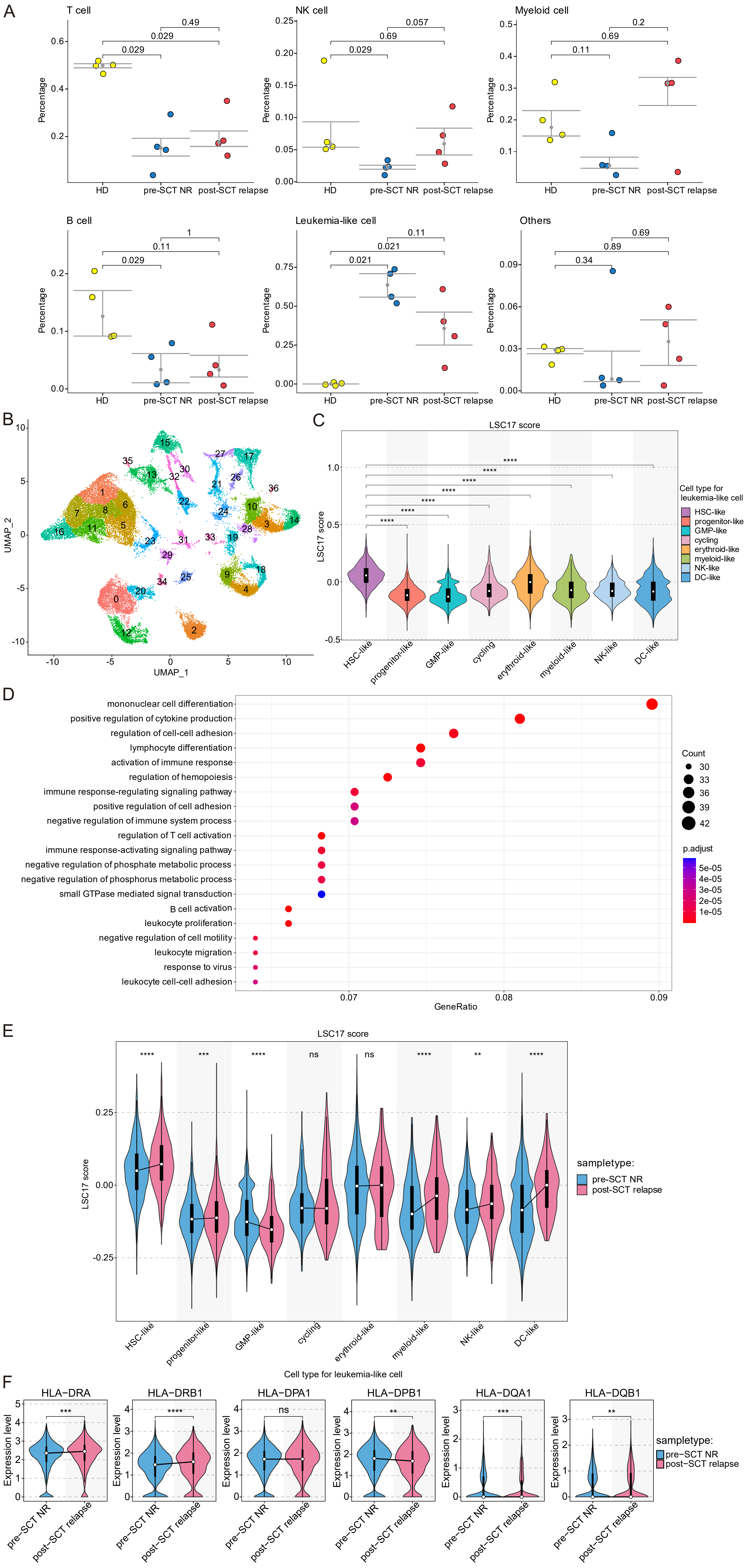

A
T cell
NK cell
Myeloid cell
B cell
Leukemia-like cell
Others
Percentage
HD
pre-SCT NR
post-SCT relapse
B
UMAP_1
UMAP_2
C
LSC17 score
Cell type for leukemia-like cell
HSC-like
progenitor-like
GMP-like
cycling
erythroid-like
myeloid-like
NK-like
DC-like
D
mononuclear cell differentiation
positive regulation of cytokine production
regulation of cell-cell adhesion
lymphocyte differentiation
activation of immune response
regulation of hemopoiesis
immune response-regulating signaling pathway
positive regulation of cell adhesion
negative regulation of immune system process
regulation of T cell activation
immune response-activating signaling pathway
negative regulation of phosphate metabolic process
negative regulation of phosphorus metabolic process
small GTPase mediated signal transduction
B cell activation
leukocyte proliferation
negative regulation of cell motility
leukocyte migration
response to virus
leukocyte cell-cell adhesion
GeneRatio
Count
p.adjust
E
LSC17 score
sampletype:
pre-SCT NR
post-SCT relapse
Cell type for leukemia-like cell
F
HLA-DRA
HLA-DRB1
HLA-DPA1
HLA-DPB1
HLA-DQA1
HLA-DQB1
Expression level
sampletype:
pre−SCT NR
post−SCT relapse

Figure S2. Characteristics of leukemia-like cells in pre-SCT NR and post-SCT relapse.

A Proportion (% of total cells) of main cell types in HD, pre-SCT NR, and post-SCT relapse sampletypes. Two-sided Wilcoxon rank sum test was used for statistical analysis. The grey points indicate the group median, the lower and upper grey errorbars represent the 25th and 75th quantiles, respectively. n = 4 independent samples in each group.

B UMAP plot of scRNA-seq data from all 37,134 leukemia-like cells colored by cluster from initial high-resolution Seurat clustering.

C Violin plot showing the LSC17 score of leukemia-like cell subtypes, comparing HSC-like blasts with other types of blasts.

D GO biological process enrichment analysis of highly expressed genes (log2FC > 0.25 and p.adj < 0.05 in Supplementary Data 3) in HSC-like blasts compared with non HSC-like blasts. GO terms with corrected $P < 0.05$ were considered significantly enriched for marker genes. Terms were sorted by the Count number, and top 20 terms were selected.

E Violin plot showing the LSC17 score of each leukemia-like cell subtype, comparing pre-SCT NR sampletype with post-SCT relapse sampletype.

F Violin plot showing the expression levels of HLA class II molecules in HSC-like blasts, comparing pre-SCT NR sampletype with post-SCT relapse sampletype.

For violin plot (**C**, **E** and **F**), the white dots inside each violin plot represent the median. The black box bodies inside each violin plot represent the interquartile ranges (IQR), the upper and lower whiskers extend from the box body to the largest and the smallest value within 1.5 times the IQR, respectively. $^{**}P < 0.01$, $^{***}P < 0.001$, $^{****}P < 0.0001$, ns, no significance. Wilcoxon rank sum test (two-sided).

Figure S3

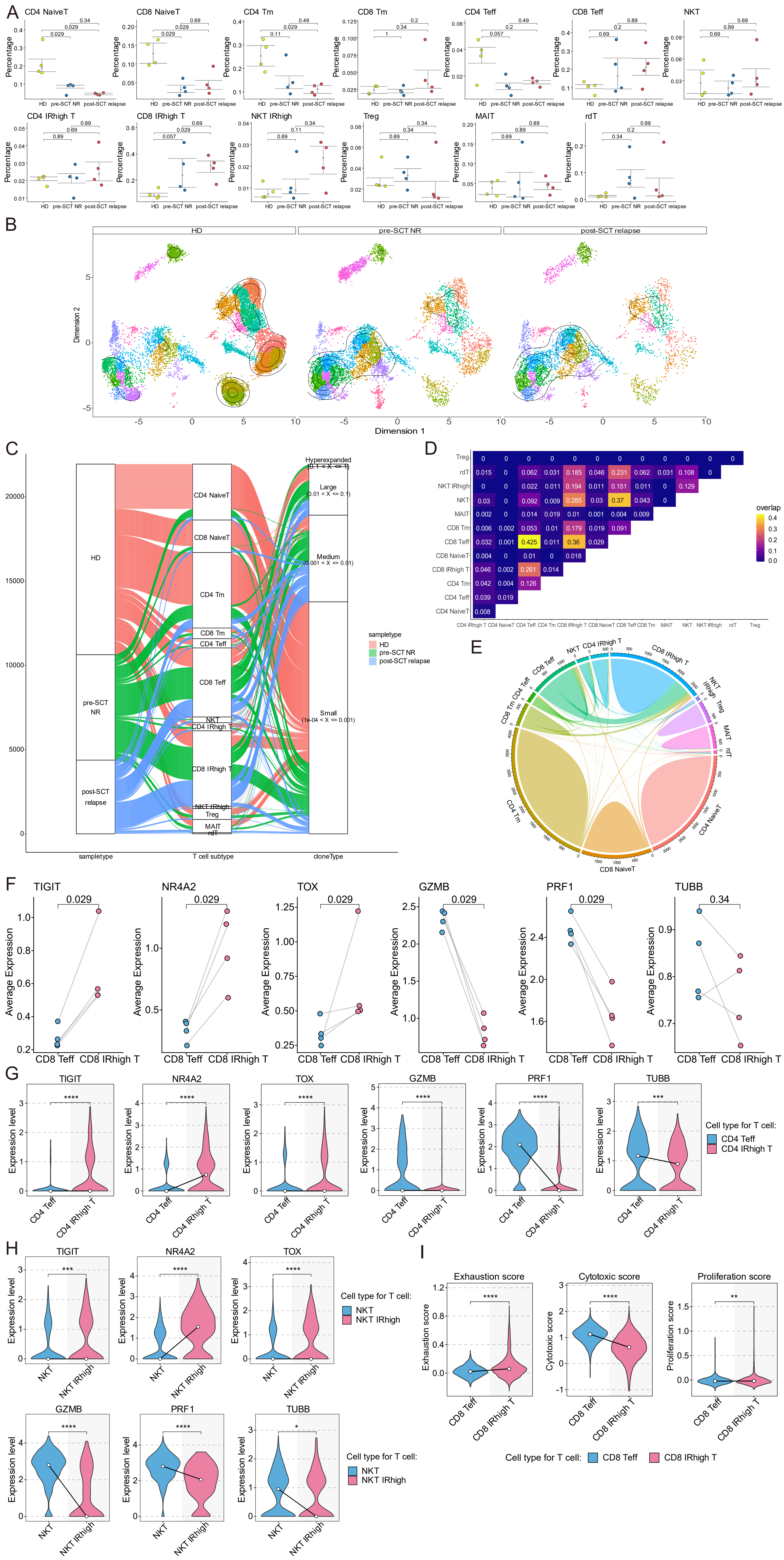
A
CD4 NaiveT
CD8 NaiveT
CD4 Tm
CD8 Tm
CD4 Teff
CD8 Teff
NKT
CD4 IRhigh T
CD8 IRhigh T
NKT IRhigh
Treg
MAIT
rdT
Percentage
HD
pre-SCT NR
post-SCT relapse
B
HD
pre-SCT NR
post-SCT relapse
Dimension 2
Dimension 1
C
sampletype
T cell subtype
cloneType
Hyperexpanded (0.1 < X <= 1)
Large (0.01 < X <= 0.1)
Medium (0.001 < X <= 0.01)
Small (1e-04 < X <= 0.001)
D
overlap
E
CD8 NaiveT
F
TIGIT
NR4A2
TOX
GZMB
PRF1
TUBB
Average Expression
CD8 Teff
CD8 IRhigh T
G
Expression level
Cell type for T cell:
CD4 Teff
CD4 IRhigh T
H
NKT
NKT IRhigh
I
Exhaustion score
Cytotoxic score
Proliferation score
Cell type for T cell:
CD8 Teff
CD8 IRhigh T

Figure S3. Single-cell transcriptomic and single-cell TCR atlas of T cells in AML patients.

A Proportion (% of total cells) of main T cell subtypes in HD, pre-SCT NR, and post-SCT relapse sampletypes. Two-sided Wilcoxon rank sum test was used for statistical analysis. The grey points indicate the group median, the lower and upper grey errorbars represent the 25th and 75th quantiles, respectively. n = 4 independent samples in each group.

B UMAP plot showing the clonal expansion location through overlaying the cells onto the dimensional reduction plots in HD, pre-SCT NR, and post-SCT relapse sampletypes. The overlay cutpoint to include was 0.0001.

C Alluvial diagram showing proportional contribution of different T cell subtypes and clonotypes corresponding to HD, pre-SCT NR, and post-SCT relapse sampletypes.

D Clonotypes overlap quantifications by subtypes in T cells.

E Chord diagram visualization of interconnection information between T cell subtypes.

F Average expression of selected marker genes representing exhaustion, cytotoxicity, and proliferation in $CD8^+$ Teff cells and $CD8^+$ IRhigh T cells. Each dot represents the mean expression of the indicated gene across cells within an individual patient. Lines connect paired samples from the same patient. Data are presented as patient-level mean expression values (n = 4 independent patients per group). A two-sided Wilcoxon signed-rank test was used for statistical analysis.

G Violin plot showing the expression level of selected marker genes representing exhaustion, cytotoxicity, and proliferation, comparing $CD4^+$ effector T (Teff) cell with $CD4^+$ IRhigh T cell.

H Violin plot showing the expression level of selected marker genes representing exhaustion, cytotoxicity, and proliferation, comparing NKT cell with NKT IRhigh cell.

I Violin plot showing the Exhaustion score, Cytotoxic score, and Proliferation score, comparing $CD8^+$ Teff cell with $CD8^+$ IRhigh T cell.

For violin plot (**G**, **H**, and **I**), the white dots inside each violin plot represent the median. *$P < 0.05$, **$P < 0.01$, ***$P < 0.001$, ****$P < 0.0001$. Wilcoxon rank sum test (two-sided).

Figure S4

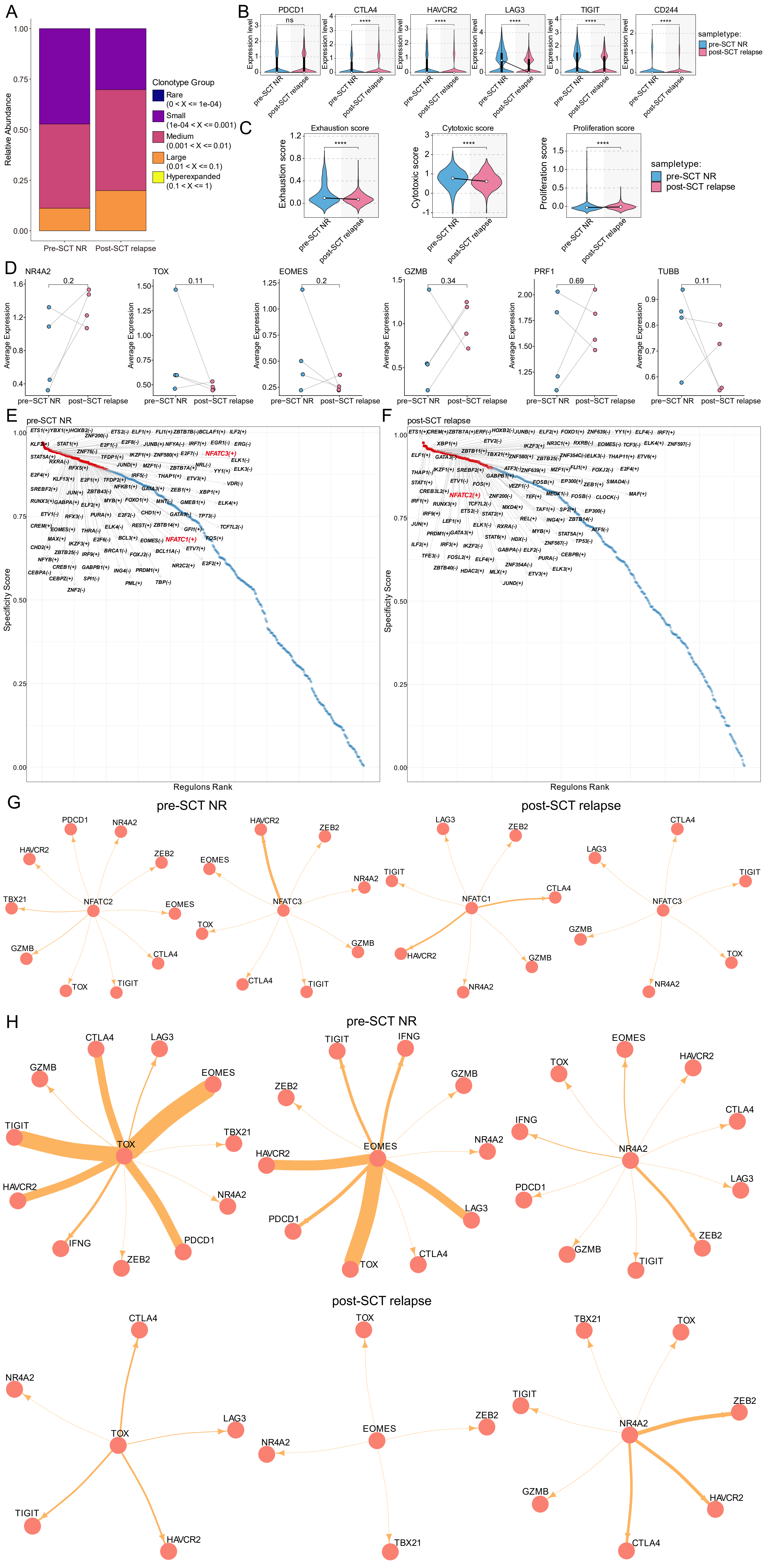

A
Relative Abundance
Pre-SCT NR
Post-SCT relapse
Clonotype Group
Rare (0 < X <= 1e-04)
Small (1e-04 < X <= 0.001)
Medium (0.001 < X <= 0.01)
Large (0.01 < X <= 0.1)
Hyperexpanded (0.1 < X <= 1)
B
PDCD1
CTLA4
HAVCR2
LAG3
TIGIT
CD244
Expression level
ns
****
pre-SCT NR
post-SCT relapse
sampletype:
pre-SCT NR
post-SCT relapse
C
Exhaustion score
Cytotoxic score
Proliferation score
D
NR4A2
TOX
EOMES
GZMB
PRF1
TUBB
Average Expression
E
pre-SCT NR
NFATC3(+)
NFATC1(+)
Specificity Score
Regulons Rank
F
post-SCT relapse
NFATC2(+)
Specificity Score
Regulons Rank
G
pre-SCT NR
post-SCT relapse
NFATC2
NFATC3
NFATC1
H
pre-SCT NR
post-SCT relapse
TOX
EOMES
NR4A2

Figure S4. Comparison of characteristics of CD8$^+$ exhausted T cells in AML patients pre- and post-transplantation.

A Clonal homeostatic space representations (clonal space occupied by clonotypes of specific proportions) of TCRs across pre-SCT NR and post-SCT relapse sampletypes.

B Violin plot showing the expression levels of selected inhibitory receptors comparing pre-SCT NR sampletype with post-SCT relapse sampletype in CD8$^+$ exhausted T cells (T_{ex}).

C Violin plot showing the Exhaustion score, Cytotoxic score, and Proliferation score comparing pre-SCT NR sampletype with post-SCT relapse sampletype in CD8$^+$ T_{ex}.

D Average expression of selected marker genes representing exhaustion, cytotoxicity, and proliferation in CD8$^+$ T_{ex} cells from pre-SCT NR and post-SCT relapse sample types. Each dot represents the mean expression of the indicated gene across cells within an individual patient. Lines connect paired samples from the same patient. Data are presented as patient-level mean expression values (n = 4 independent patients per group). A two-sided Wilcoxon signed-rank test was used for statistical analysis.

E Top 100 regulons of pre-SCT NR sampletype in CD8$^+$ T_{ex}.

F Top 100 regulons of post-SCT relapse sampletype in CD8$^+$ T_{ex}.

G Gene regulatory network of transcription factor NFATC2, NFATC3 in pre-SCT NR sampletype and NFATC1, NFATC3 in post-SCT relapse sampletype in CD8$^+$ T_{ex}.

H Gene regulatory network of transcription factor TOX, EOMES, and NR4A2 in pre-SCT NR and post-SCT relapse sampletypes in CD8$^+$ T_{ex}.

For violin plots (**B** and **C**), the white dot inside each violin plot represents the median. The black box body inside each violin plot represents the interquartile range (IQR); the upper and lower whiskers extend from the box body to the largest and smallest values within 1.5 times the IQR, respectively. $^{****}P < 0.0001$; ns, not significant. Wilcoxon rank sum test (two-sided).

For gene regulatory network plots (**G** and **H**), the direction of the arrow represents the direction of gene regulation, and the thickness of the line represents the weight of regulation.

Figure S5

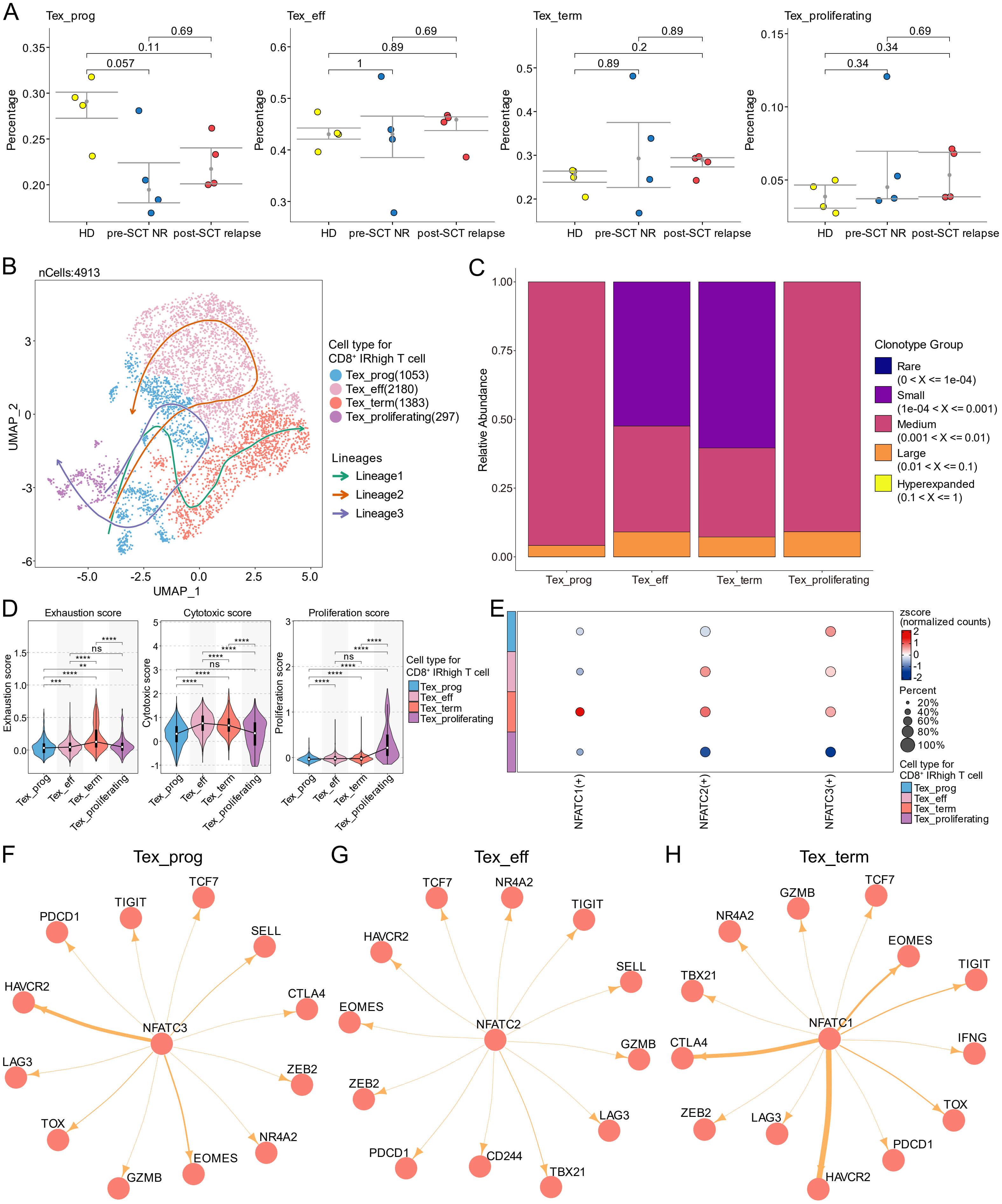

A
Tex_prog
Tex_eff
Tex_term
Tex_proliferating
Percentage
HD
pre-SCT NR
post-SCT relapse
0.057
0.11
0.69
1
0.89
0.2
0.34
B
nCells:4913
UMAP_1
UMAP_2
Cell type for
CD8+ IRhigh T cell
Tex_prog(1053)
Tex_eff(2180)
Tex_term(1383)
Tex_proliferating(297)
Lineages
Lineage1
Lineage2
Lineage3
C
Relative Abundance
Clonotype Group
Rare
(0 < X <= 1e-04)
Small
(1e-04 < X <= 0.001)
Medium
(0.001 < X <= 0.01)
Large
(0.01 < X <= 0.1)
Hyperexpanded
(0.1 < X <= 1)
D
Exhaustion score
Cytotoxic score
Proliferation score
Cell type for
CD8+ IRhigh T cell
Tex_prog
Tex_eff
Tex_term
Tex_proliferating
E
zscore
(normalized counts)
Percent
20%
40%
60%
80%
100%
NFATC1(+)
NFATC2(+)
NFATC3(+)
F
Tex_prog
NFATC3
PDCD1
TIGIT
TCF7
SELL
HAVCR2
CTLA4
LAG3
ZEB2
TOX
NR4A2
EOMES
GZMB
G
Tex_eff
NFATC2
TCF7
NR4A2
TIGIT
HAVCR2
SELL
EOMES
GZMB
ZEB2
LAG3
PDCD1
CD244
TBX21
H
Tex_term
NFATC1
GZMB
TCF7
NR4A2
EOMES
TBX21
TIGIT
CTLA4
IFNG
ZEB2
LAG3
TOX
PDCD1
HAVCR2

Figure S5. Comparison of characteristics of CD8$^+$ exhausted T cell subtypes in AML patients pre- and post-transplantation.

A Proportion (% of total cells) of main CD8$^+$ T_{ex} subtypes in HD, pre-SCT NR, and post-SCT relapse sampletypes. Two-sided Wilcoxon rank sum test was used for statistical analysis. The grey points indicate the group median, the lower and upper grey errorbars represent the 25th and 75th quantiles, respectively. n = 4 independent samples in each group.

B UMAP plot of CD8$^+$ T_{ex} colored by CD8$^+$ T_{ex} subtypes with Slingshot trajectories overlaid.

C Clonal homeostatic space representations (clonal space occupied by clonotypes of specific proportions) of TCRs across different CD8$^+$ T_{ex} subtypes.

D Violin plot showing the Exhaustion score, Cytotoxic score, and Proliferation score comparing between different CD8$^+$ T_{ex} subtypes. The white dot inside each violin plot represents the median. The black box body inside each violin plot represents the interquartile ranges (IQR), the upper and lower whiskers extend from the box body to the largest and the smallest value within 1.5 times the IQR, respectively. $**P < 0.01$, $***P < 0.001$, $****P < 0.0001$, ns, no significance. Wilcoxon rank sum test (two-sided).

E Dot plot showing activation of selected TF motifs evaluated by pySCENIC for different CD8$^+$ T_{ex} subtypes. The colors represent the normalized AUC of selected TF motifs, and dot sizes represent the percentage activity of selected TF motifs in the indicated clusters.

F Gene regulatory network of transcription factor NFATC3 in progenitor T_{ex} (T_{ex}_prog) cell.

G Gene regulatory network of transcription factor NFATC2 in effector T_{ex} (T_{ex}_eff) cell.

H Gene regulatory network of transcription factor NFATC1 in terminally T_{ex} (T_{ex}_term) cell.

For gene regulatory network plots (**F**, **G**, and **H**), the direction of the arrow represents the direction of gene regulation, and the thickness of the line represents the weight of regulation.

Figure S6

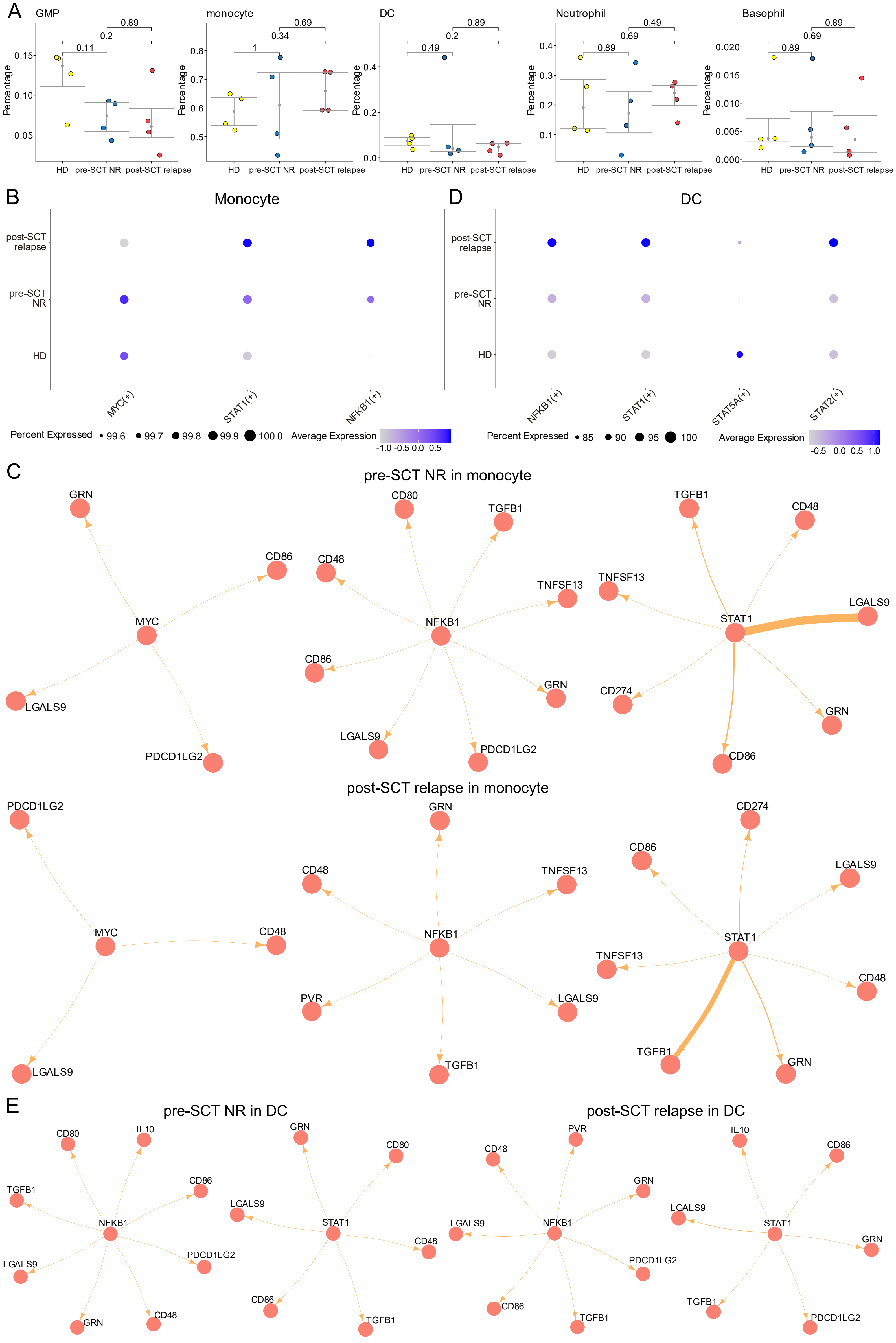

A
GMP
monocyte
DC
Neutrophil
Basophil
Percentage
HD
pre-SCT NR
post-SCT relapse
B
Monocyte
D
DC
Percent Expressed
Average Expression
MYC(+)
STAT1(+)
NFKB1(+)
STAT5A(+)
STAT2(+)
C
pre-SCT NR in monocyte
post-SCT relapse in monocyte
E
pre-SCT NR in DC
post-SCT relapse in DC
MYC
NFKB1
STAT1
GRN
CD80
TGFB1
CD48
CD86
TNFSF13
LGALS9
PDCD1LG2
CD274
PVR
IL10

Figure S6. Comparison among different sampletypes of characteristics of myeloid cells and immune microenvironment.

A Proportion (% of total cells) of main myeloid cell subtypes in HD, pre-SCT NR, and post-SCT relapse sampletypes. Two-sided Wilcoxon rank sum test was used for statistical analysis. The grey points indicate the group median, the lower and upper grey errorbars represent the 25th and 75th quantiles, respectively. n = 4 independent samples in each group.

B Dot plot showing activation of selected TF motifs evaluated by pySCENIC for HD, pre-SCT NR and post-SCT relapse sampletypes in monocyte.

C Gene regulatory network of transcription factor MYC, NFKB1, and STAT1 for pre-SCT NR and post-SCT relapse sampletypes in monocyte.

D Dot plot showing activation of selected TF motifs evaluated by pySCENIC for HD, pre-SCT NR and post-SCT relapse sampletypes in DC.

E Gene regulatory network of transcription factor NFKB1 and STAT1 for pre-SCT NR and post-SCT relapse sampletypes in DC.

For dot plots (**B** and **D**), the colors represent the normalized AUC of selected TF motifs, and dot sizes represent the percentage activity of selected TF motifs in the indicated clusters.

For gene regulatory network plots (**C** and **E**), the direction of the arrow represents the direction of gene regulation, and the thickness of the line represents the weight of regulation.

Figure S7

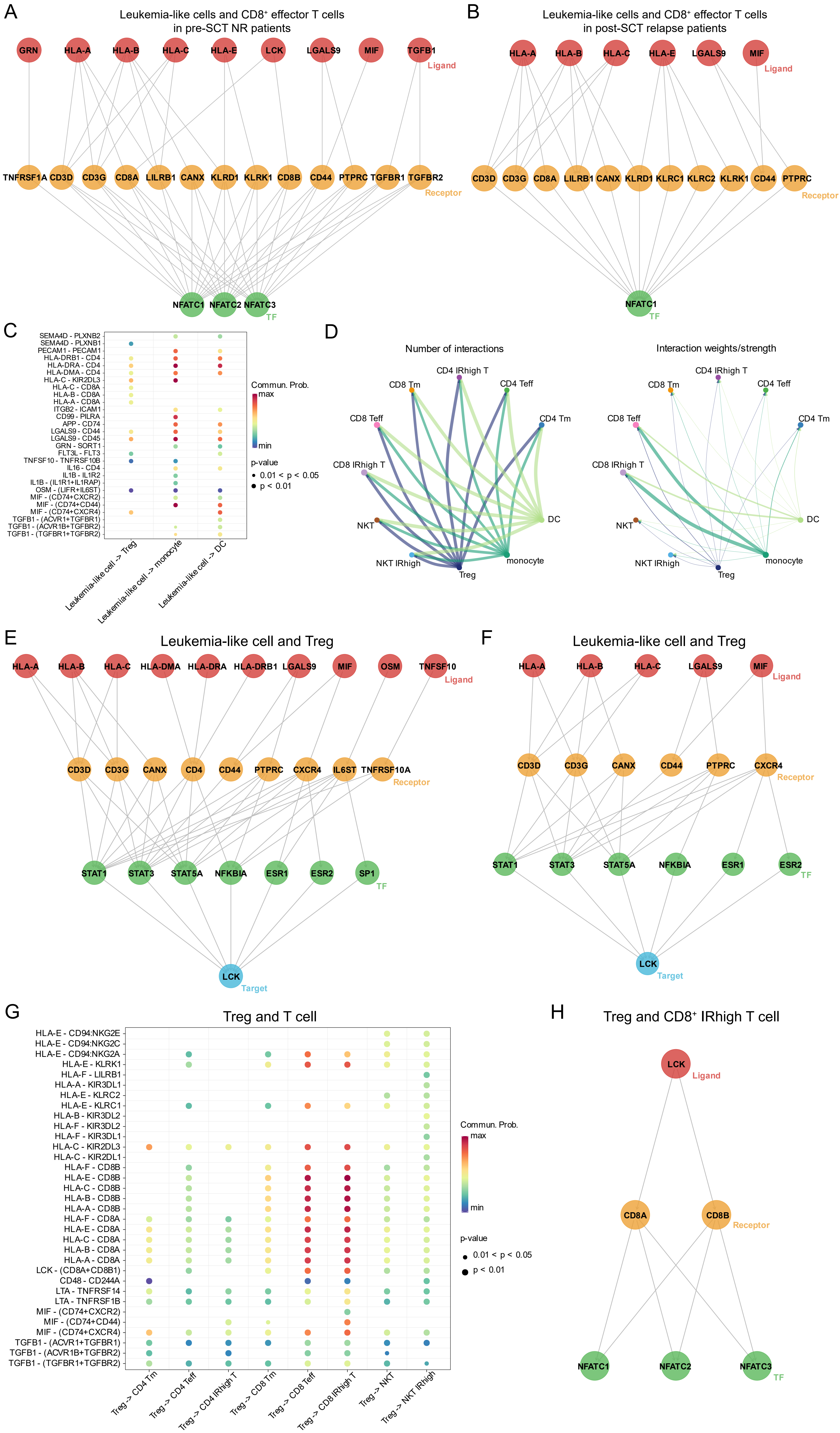
A
Leukemia-like cells and CD8⁺ effector T cells in pre-SCT NR patients
GRN HLA-A HLA-B HLA-C HLA-E LCK LGALS9 MIF TGFB1
Ligand
TNFRSF1A CD3D CD3G CD8A LILRB1 CANX KLRD1 KLRK1 CD8B CD44 PTPRC TGFBR1 TGFBR2
Receptor
NFATC1 NFATC2 NFATC3
TF
B
Leukemia-like cells and CD8⁺ effector T cells in post-SCT relapse patients
HLA-A HLA-B HLA-C HLA-E LGALS9 MIF
Ligand
CD3D CD3G CD8A LILRB1 CANX KLRD1 KLRC1 KLRC2 KLRK1 CD44 PTPRC
Receptor
NFATC1
TF
C
SEMA4D - PLXNB2
SEMA4D - PLXNB1
PECAM1 - PECAM1
HLA-DRB1 - CD4
HLA-DRA - CD4
HLA-DMA - CD4
HLA-C - KIR2DL3
HLA-C - CD8A
HLA-B - CD8A
HLA-A - CD8A
ITGB2 - ICAM1
CD99 - PILRA
APP - CD74
LGALS9 - CD44
LGALS9 - CD45
GRN - SORT1
FLT3L - FLT3
TNFSF10 - TNFRSF10B
IL16 - CD4
IL1B - IL1R2
IL1B - (IL1R1+IL1RAP)
OSM - (LIFR+IL6ST)
MIF - (CD74+CXCR2)
MIF - (CD74+CD44)
MIF - (CD74+CXCR4)
TGFB1 - (ACVR1+TGFBR1)
TGFB1 - (ACVR1B+TGFBR2)
TGFB1 - (TGFBR1+TGFBR2)
Leukemia-like cell -> Treg
Leukemia-like cell -> monocyte
Leukemia-like cell -> DC
Commun. Prob.
max
min
p-value
0.01 < p < 0.05
p < 0.01
D
Number of interactions
Interaction weights/strength
CD4 IRhigh T
CD8 Tm
CD4 Teff
CD8 Teff
CD4 Tm
CD8 IRhigh T
DC
NKT
monocyte
NKT IRhigh
Treg
E
Leukemia-like cell and Treg
HLA-A HLA-B HLA-C HLA-DMA HLA-DRA HLA-DRB1 LGALS9 MIF OSM TNFSF10
Ligand
CD3D CD3G CANX CD4 CD44 PTPRC CXCR4 IL6ST TNFRSF10A
Receptor
STAT1 STAT3 STAT5A NFKBIA ESR1 ESR2 SP1
TF
LCK
Target
F
Leukemia-like cell and Treg
HLA-A HLA-B HLA-C LGALS9 MIF
Ligand
CD3D CD3G CANX CD44 PTPRC CXCR4
Receptor
STAT1 STAT3 STAT5A NFKBIA ESR1 ESR2
TF
LCK
Target
G
Treg and T cell
HLA-E - CD94:NKG2E
HLA-E - CD94:NKG2C
HLA-E - CD94:NKG2A
HLA-E - KLRK1
HLA-F - LILRB1
HLA-A - KIR3DL1
HLA-E - KLRC2
HLA-E - KLRC1
HLA-B - KIR3DL2
HLA-F - KIR3DL2
HLA-F - KIR3DL1
HLA-C - KIR2DL3
HLA-C - KIR2DL1
HLA-F - CD8B
HLA-E - CD8B
HLA-C - CD8B
HLA-B - CD8B
HLA-A - CD8B
HLA-F - CD8A
HLA-E - CD8A
HLA-C - CD8A
HLA-B - CD8A
HLA-A - CD8A
LCK - (CD8A+CD8B1)
CD48 - CD244A
LTA - TNFRSF14
LTA - TNFRSF1B
MIF - (CD74+CXCR2)
MIF - (CD74+CD44)
MIF - (CD74+CXCR4)
TGFB1 - (ACVR1+TGFBR1)
TGFB1 - (ACVR1B+TGFBR2)
TGFB1 - (TGFBR1+TGFBR2)
Treg -> CD4 Tm
Treg -> CD4 Teff
Treg -> CD4 IRhigh T
Treg -> CD8 Tm
Treg -> CD8 Teff
Treg -> CD8 IRhigh T
Treg -> NKT
Treg -> NKT IRhigh
Commun. Prob.
max
min
p-value
0.01 < p < 0.05
p < 0.01
H
Treg and CD8⁺ IRhigh T cell
LCK
Ligand
CD8A CD8B
Receptor
NFATC1 NFATC2 NFATC3
TF

Figure S7. Interactions between leukemia-like cells and CD8$^+$ effector T cells in both pre-SCT NR and post-SCT relapse patients and interactions among leukemia-like cells, Tregs, and T cells in pre-SCT NR AML patients.

A The molecular regulatory network between leukemia-like cells and CD8$^+$ effector T cells in pre-SCT NR patients. The multilayer network contained three layers of ligand, receptor, and TF, which were marked by different colors.

B The molecular regulatory network between leukemia-like cells and CD8$^+$ effector T cells in post-SCT relapse patients. The multilayer network contained three layers of ligand, receptor, and TF, which were marked by different colors.

C Dot plot showing the intersections of significant functional ligand-receptor pairs that contribute to the signaling sending from leukemia-like cell to Treg, monocyte, and DC in pre-SCT NR patients.

D The number of interaction (left) and the weight of interaction (right) of Treg, monocyte, and DC in pre-SCT NR patients. Different colors represent different cell groups, and the thickness of the lines represents the number of interactions in count plot or the weights/strength of interactions in weight plot between the two cell types.

E The molecular regulatory network between leukemia-like cells and Treg in pre-SCT NR patients. The multilayer network contained four layers of ligand, receptor, TF, and target, which were marked by different colors. The target was the intersection of ligands involved in the interaction between Treg and CD8$^+$ T_{ex}.

F The molecular regulatory network between leukemia-like cells and Treg in pre-SCT NR patients. The multilayer network contained four layers of ligand, receptor, TF, and target, which were marked by different colors. The ligands were the intersection of ligands involved in the interaction between leukemia-like cells and CD8$^+$ T_{ex}, and the target was the intersection of ligands involved in the interaction between Treg and CD8$^+$ T_{ex}.

G Dot plot showing the intersections of significant functional ligand-receptor pairs that contribute to the signaling sending from Treg to eight T cell subtypes in pre-SCT NR patients.

H The molecular regulatory network between Treg and CD8$^+$ T_{ex} in pre-SCT NR patients. The multilayer network contained three layers of ligand, receptor, and TF, which were marked by different colors. The ligand was the intersection of targets involved in the interaction between leukemia-like cells and Treg.

For dot plots (**C** and **G**), dot color represents communication probabilities, dot size represents one-sided permutation test *p*-values, and empty space indicates that the communication probability is zero.

For molecular regulatory network plots (**A**, **B**, **E**, **F**, and **H**), the ligands were the intersection between CellChat and scMLnet analysis.

Figure S8

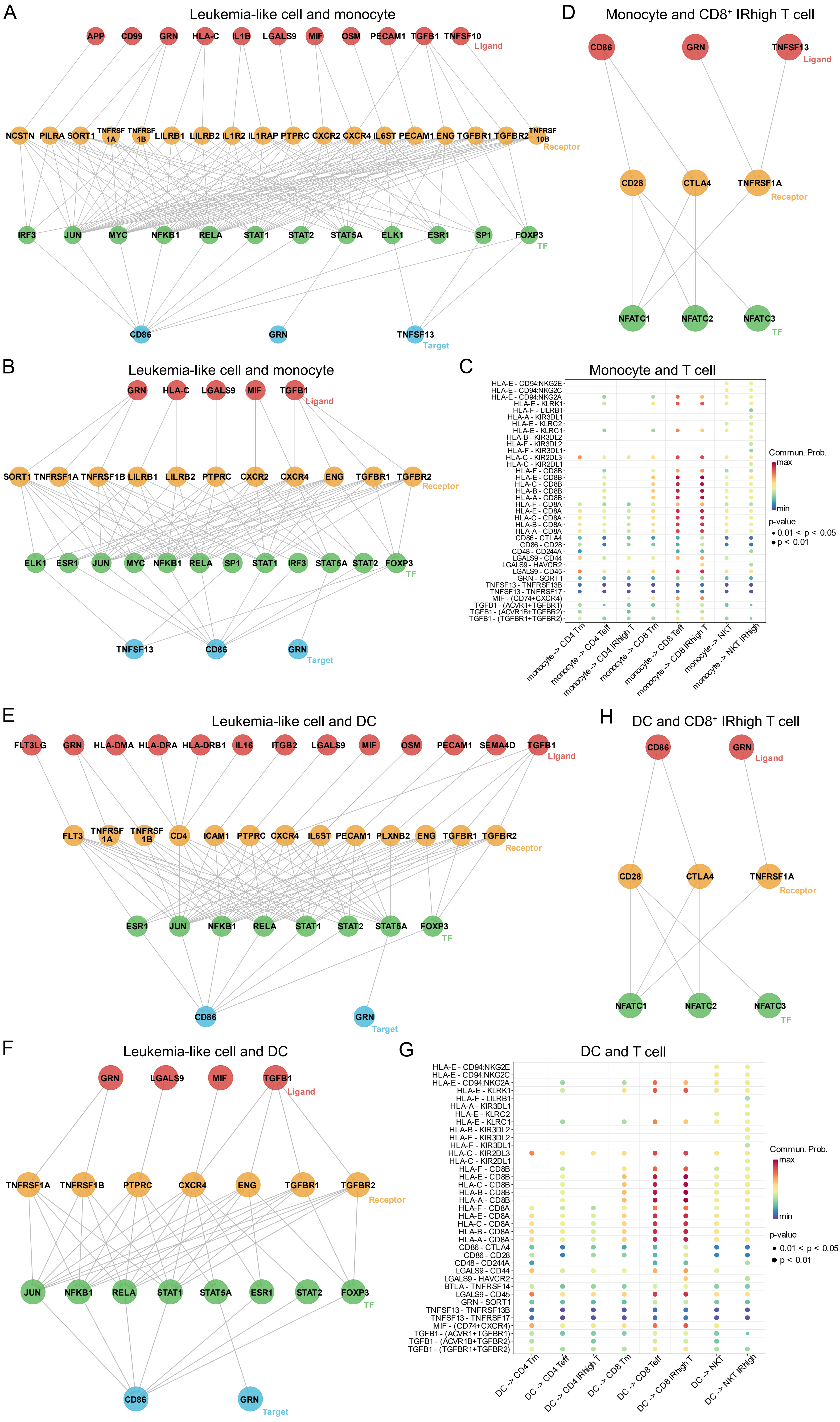
A
Leukemia-like cell and monocyte
APP CD99 GRN HLA-C IL1B LGALS9 MIF OSM PECAM1 TGFB1 TNFSF10
Ligand
NCSTN PILRA SORT1 TNFRSF 1A TNFRSF 1B LILRB1 LILRB2 IL1R2 IL1RAP PTPRC CXCR2 CXCR4 IL6ST PECAM1 ENG TGFBR1 TGFBR2 TNFRSF 10B
Receptor
IRF3 JUN MYC NFKB1 RELA STAT1 STAT2 STAT5A ELK1 ESR1 SP1 FOXP3
TF
CD86 GRN TNFSF13
Target
D
Monocyte and CD8+ IRhigh T cell
CD86 GRN TNFSF13
Ligand
CD28 CTLA4 TNFRSF1A
Receptor
NFATC1 NFATC2 NFATC3
TF
B
Leukemia-like cell and monocyte
GRN HLA-C LGALS9 MIF TGFB1
Ligand
SORT1 TNFRSF1A TNFRSF1B LILRB1 LILRB2 PTPRC CXCR2 CXCR4 ENG TGFBR1 TGFBR2
Receptor
ELK1 ESR1 JUN MYC NFKB1 RELA SP1 STAT1 IRF3 STAT5A STAT2 FOXP3
TF
TNFSF13 CD86 GRN
Target
C
Monocyte and T cell
HLA-E - CD94:NKG2E
HLA-E - CD94:NKG2C
HLA-E - CD94:NKG2A
HLA-E - KLRK1
HLA-F - LILRB1
HLA-A - KIR3DL1
HLA-E - KLRC2
HLA-E - KLRC1
HLA-B - KIR3DL2
HLA-F - KIR3DL2
HLA-F - KIR3DL1
HLA-C - KIR2DL3
HLA-C - KIR2DL1
HLA-F - CD8B
HLA-E - CD8B
HLA-C - CD8B
HLA-B - CD8B
HLA-A - CD8B
HLA-F - CD8A
HLA-E - CD8A
HLA-C - CD8A
HLA-B - CD8A
HLA-A - CD8A
CD86 - CTLA4
CD86 - CD28
CD48 - CD244A
LGALS9 - CD44
LGALS9 - HAVCR2
LGALS9 - CD45
GRN - SORT1
TNFSF13 - TNFRSF13B
TNFSF13 - TNFRSF17
MIF - (CD74+CXCR4)
TGFB1 - (ACVR1+TGFBR1)
TGFB1 - (ACVR1B+TGFBR2)
TGFB1 - (TGFBR1+TGFBR2)
monocyte -> CD4 Tm
monocyte -> CD4 Teff
monocyte -> CD4 IRhigh T
monocyte -> CD8 Tm
monocyte -> CD8 Teff
monocyte -> CD8 IRhigh T
monocyte -> NKT
monocyte -> NKT IRhigh
Commun. Prob.
max
min
p-value
0.01 < p < 0.05
p < 0.01
E
Leukemia-like cell and DC
FLT3LG GRN HLA-DMA HLA-DRA HLA-DRB1 IL16 ITGB2 LGALS9 MIF OSM PECAM1 SEMA4D TGFB1
Ligand
FLT3 TNFRSF 1A TNFRSF 1B CD4 ICAM1 PTPRC CXCR4 IL6ST PECAM1 PLXNB2 ENG TGFBR1 TGFBR2
Receptor
ESR1 JUN NFKB1 RELA STAT1 STAT2 STAT5A FOXP3
TF
CD86 GRN
Target
H
DC and CD8+ IRhigh T cell
CD86 GRN
Ligand
CD28 CTLA4 TNFRSF1A
Receptor
NFATC1 NFATC2 NFATC3
TF
F
Leukemia-like cell and DC
GRN LGALS9 MIF TGFB1
Ligand
TNFRSF1A TNFRSF1B PTPRC CXCR4 ENG TGFBR1 TGFBR2
Receptor
JUN NFKB1 RELA STAT1 STAT5A ESR1 STAT2 FOXP3
TF
CD86 GRN
Target
G
DC and T cell
HLA-E - CD94:NKG2E
HLA-E - CD94:NKG2C
HLA-E - CD94:NKG2A
HLA-E - KLRK1
HLA-F - LILRB1
HLA-A - KIR3DL1
HLA-E - KLRC2
HLA-E - KLRC1
HLA-B - KIR3DL2
HLA-F - KIR3DL2
HLA-F - KIR3DL1
HLA-C - KIR2DL3
HLA-C - KIR2DL1
HLA-F - CD8B
HLA-E - CD8B
HLA-C - CD8B
HLA-B - CD8B
HLA-A - CD8B
HLA-F - CD8A
HLA-E - CD8A
HLA-C - CD8A
HLA-B - CD8A
HLA-A - CD8A
CD86 - CTLA4
CD86 - CD28
CD48 - CD244A
LGALS9 - CD44
LGALS9 - HAVCR2
BTLA - TNFRSF14
LGALS9 - CD45
GRN - SORT1
TNFSF13 - TNFRSF13B
TNFSF13 - TNFRSF17
MIF - (CD74+CXCR4)
TGFB1 - (ACVR1+TGFBR1)
TGFB1 - (ACVR1B+TGFBR2)
TGFB1 - (TGFBR1+TGFBR2)
DC -> CD4 Tm
DC -> CD4 Teff
DC -> CD4 IRhigh T
DC -> CD8 Tm
DC -> CD8 Teff
DC -> CD8 IRhigh T
DC -> NKT
DC -> NKT IRhigh
Commun. Prob.
max
min
p-value
0.01 < p < 0.05
p < 0.01

Figure S8. Interactions among leukemia-like cells, monocytes, DCs, and T cells in pre-SCT NR AML patients.

A The molecular regulatory network between leukemia-like cells and monocyte. The multilayer network contained four layers of ligand, receptor, TF, and target, which were marked by different colors. The targets were the intersection of ligands involved in the interaction between monocyte and $CD8^+$ T_{ex}.

B The molecular regulatory network between leukemia-like cells and monocyte. The multilayer network contained four layers of ligand, receptor, TF, and target, which were marked by different colors. The ligands were the intersection of ligands involved in the interaction between leukemia-like cells and $CD8^+$ T_{ex}, and the targets were the intersection of ligands involved in the interaction between monocyte and $CD8^+$ T_{ex}.

C Dot plot showing the intersections of significant functional ligand-receptor pairs that contribute to the signaling sending from monocyte to eight T cell subtypes.

D The molecular regulatory network between monocyte and $CD8^+$ T_{ex}. The multilayer network contained three layers of ligand, receptor, and TF, which were marked by different colors. The ligands were the intersection of targets involved in the interaction between leukemia-like cells and monocyte.

E The molecular regulatory network between leukemia-like cells and DC. The multilayer network contained four layers of ligand, receptor, TF, and target, which were marked by different colors. The targets were the intersection of ligands involved in the interaction between DC and $CD8^+$ T_{ex}.

F The molecular regulatory network between leukemia-like cells and DC. The multilayer network contained four layers of ligand, receptor, TF, and target, which were marked by different colors. The ligands were the intersection of ligands involved in the interaction between leukemia-like cells and $CD8^+$ T_{ex}, and the targets were the intersection of ligands involved in the interaction between DC and $CD8^+$ T_{ex}.

G Dot plot showing the intersections of significant functional ligand-receptor pairs that contribute to the signaling sending from DC to eight T cell subtypes.

H The molecular regulatory network between DC and $CD8^+$ T_{ex}. The multilayer network contained three layers of ligand, receptor, and TF, which were marked by different colors. The ligands were the intersection of targets involved in the interaction between leukemia-like cells and DC.

For dot plots (**C** and **G**), dot color represents communication probabilities, dot size represents one-sided permutation test *p*-values, and empty space indicates that the communication probability is zero.

For molecular regulatory network plots (**A**, **B**, **D**, **E**, **F**, and **H**), the ligands were the intersection between CellChat and scMLnet analysis.

Figure S9

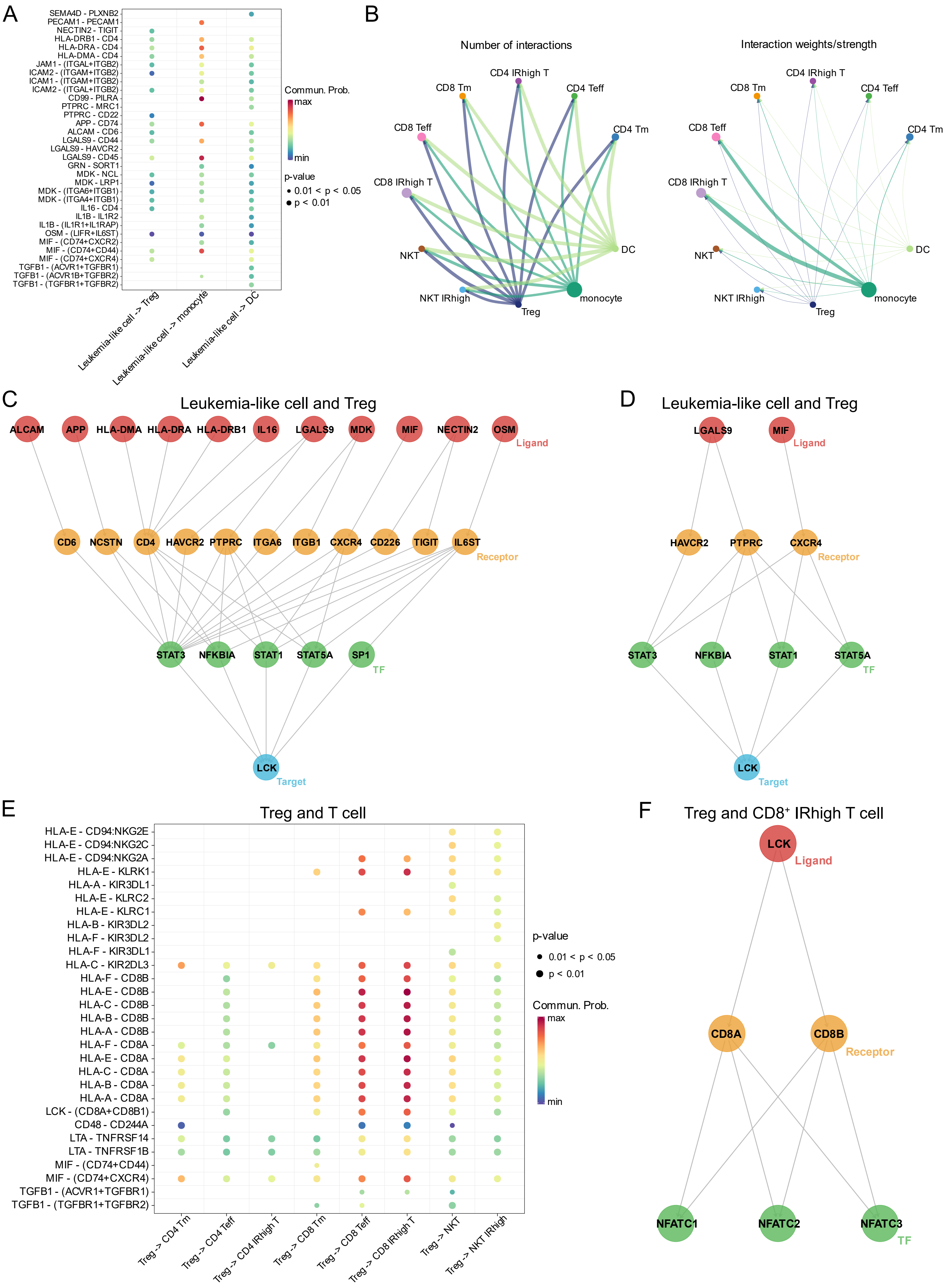
A
SEMA4D - PLXNB2
PECAM1 - PECAM1
NECTIN2 - TIGIT
HLA-DRB1 - CD4
HLA-DRA - CD4
HLA-DMA - CD4
JAM1 - (ITGAL+ITGB2)
ICAM2 - (ITGAM+ITGB2)
ICAM1 - (ITGAM+ITGB2)
ICAM2 - (ITGAL+ITGB2)
CD99 - PILRA
PTPRC - MRC1
PTPRC - CD22
APP - CD74
ALCAM - CD6
LGALS9 - CD44
LGALS9 - HAVCR2
LGALS9 - CD45
GRN - SORT1
MDK - NCL
MDK - LRP1
MDK - (ITGA6+ITGB1)
MDK - (ITGA4+ITGB1)
IL16 - CD4
IL1B - IL1R2
IL1B - (IL1R1+IL1RAP)
OSM - (LIFR+IL6ST)
MIF - (CD74+CXCR2)
MIF - (CD74+CD44)
MIF - (CD74+CXCR4)
TGFB1 - (ACVR1+TGFBR1)
TGFB1 - (ACVR1B+TGFBR2)
TGFB1 - (TGFBR1+TGFBR2)
Leukemia-like cell -> Treg
Leukemia-like cell -> monocyte
Leukemia-like cell -> DC
Commun. Prob.
max
min
p-value
0.01 < p < 0.05
p < 0.01
B
Number of interactions
Interaction weights/strength
CD4 IRhigh T
CD8 Tm
CD4 Teff
CD8 Teff
CD4 Tm
CD8 IRhigh T
NKT
DC
NKT IRhigh
Treg
monocyte
C
Leukemia-like cell and Treg
ALCAM
APP
HLA-DMA
HLA-DRA
HLA-DRB1
IL16
LGALS9
MDK
MIF
NECTIN2
OSM
Ligand
CD6
NCSTN
CD4
HAVCR2
PTPRC
ITGA6
ITGB1
CXCR4
CD226
TIGIT
IL6ST
Receptor
STAT3
NFKBIA
STAT1
STAT5A
SP1
TF
LCK
Target
D
Leukemia-like cell and Treg
LGALS9
MIF
Ligand
HAVCR2
PTPRC
CXCR4
Receptor
STAT3
NFKBIA
STAT1
STAT5A
TF
LCK
Target
E
Treg and T cell
HLA-E - CD94:NKG2E
HLA-E - CD94:NKG2C
HLA-E - CD94:NKG2A
HLA-E - KLRK1
HLA-A - KIR3DL1
HLA-E - KLRC2
HLA-E - KLRC1
HLA-B - KIR3DL2
HLA-F - KIR3DL2
HLA-F - KIR3DL1
HLA-C - KIR2DL3
HLA-F - CD8B
HLA-E - CD8B
HLA-C - CD8B
HLA-B - CD8B
HLA-A - CD8B
HLA-F - CD8A
HLA-E - CD8A
HLA-C - CD8A
HLA-B - CD8A
HLA-A - CD8A
LCK - (CD8A+CD8B1)
CD48 - CD244A
LTA - TNFRSF14
LTA - TNFRSF1B
MIF - (CD74+CD44)
MIF - (CD74+CXCR4)
TGFB1 - (ACVR1+TGFBR1)
TGFB1 - (TGFBR1+TGFBR2)
Treg -> CD4 Tm
Treg -> CD4 Teff
Treg -> CD4 IRhigh T
Treg -> CD8 Tm
Treg -> CD8 Teff
Treg -> CD8 IRhigh T
Treg -> NKT
Treg -> NKT IRhigh
p-value
0.01 < p < 0.05
p < 0.01
Commun. Prob.
max
min
F
Treg and CD8+ IRhigh T cell
LCK
Ligand
CD8A
CD8B
Receptor
NFATC1
NFATC2
NFATC3
TF

Figure S9. Interactions among leukemia-like cells, Tregs, and T cells in post-SCT relapse AML patients.

A Dot plot showing the intersections of significant functional ligand-receptor pairs that contribute to the signaling sending from leukemia-like cell to Treg, monocyte, and DC.

B The number of interaction (left) and the weight of interaction (right) of Treg, monocyte, and DC. Different colors represent different cell groups, and the thickness of the lines represents the number of interactions in count plot or the weights/strength of interactions in weight plot between the two cell types.

C The molecular regulatory network between leukemia-like cells and Treg. The multilayer network contained four layers of ligand, receptor, TF, and target, which were marked by different colors. The target was the intersection of ligands involved in the interaction between Treg and $CD8^+$ T_{ex}.

D The molecular regulatory network between leukemia-like cells and Treg. The multilayer network contained four layers of ligand, receptor, TF, and target, which were marked by different colors. The ligands were the intersection of ligands involved in the interaction between leukemia-like cells and $CD8^+$ T_{ex} in pre-SCT NR settings, and the target was the intersection of ligands involved in the interaction between Treg and $CD8^+$ T_{ex}.

E Dot plot showing the intersections of significant functional ligand-receptor pairs that contribute to the signaling sending from Treg to eight T cell subtypes.

F The molecular regulatory network between Treg and $CD8^+$ T_{ex}. The multilayer network contained three layers of ligand, receptor, and TF, which were marked by different colors. The ligand was the intersection of targets involved in the interaction between leukemia-like cells and Treg.

For dot plots (**A** and **E**), dot color represents communication probabilities, dot size represents one-sided permutation test *p*-values, and empty space indicates that the communication probability is zero.

For molecular regulatory network plots (**C**, **D**, and **F**), the ligands were the intersection between CellChat and scMLnet analysis.

Figure S10

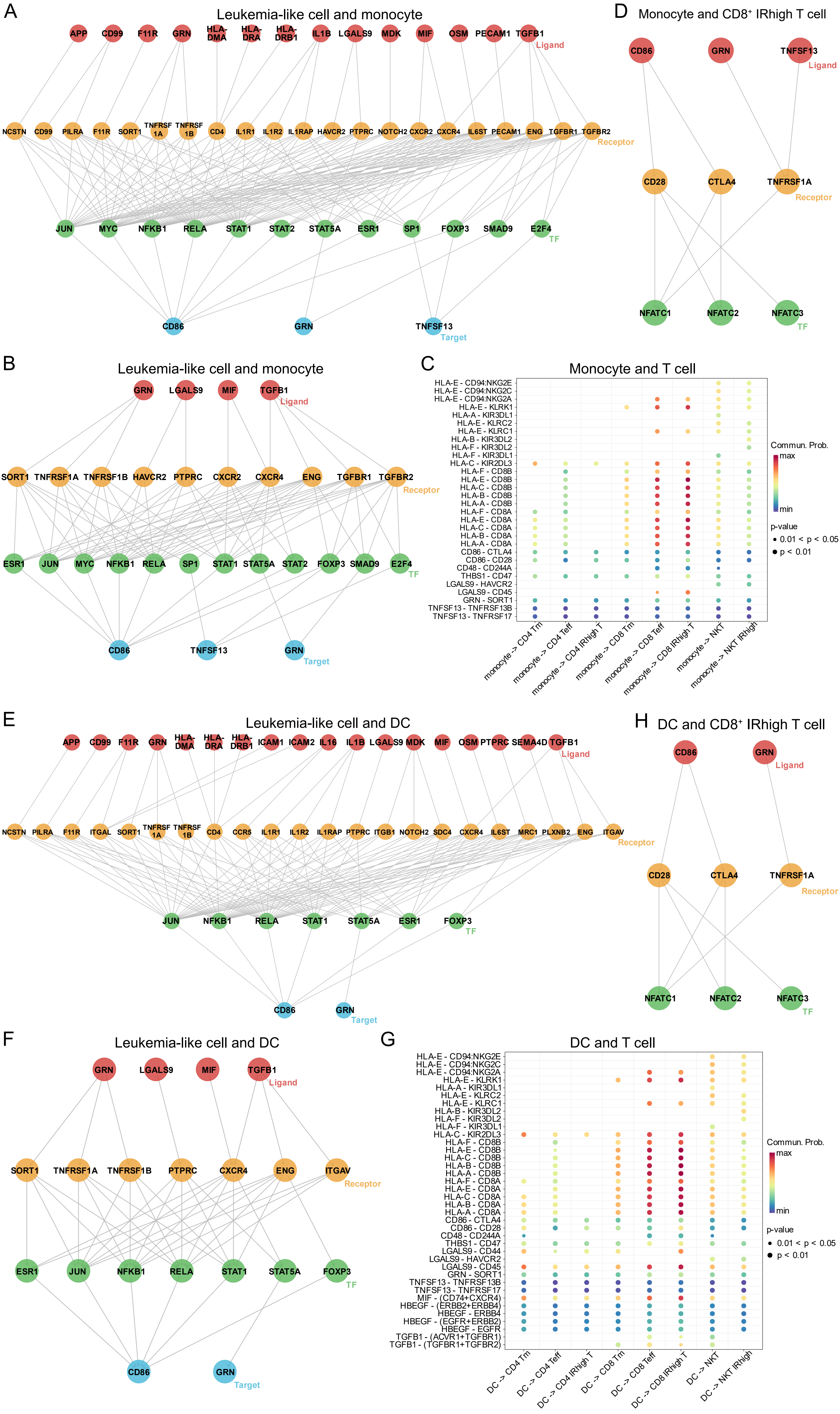
A
Leukemia-like cell and monocyte
APP CD99 F11R GRN HLA-DMA HLA-DRA HLA-DRB1 IL1B LGALS9 MDK MIF OSM PECAM1 TGFB1
Ligand
NCSTN CD99 PILRA F11R SORT1 TNFRSF1A TNFRSF1B CD4 IL1R1 IL1R2 IL1RAP HAVCR2 PTPRC NOTCH2 CXCR2 CXCR4 IL6ST PECAM1 ENG TGFBR1 TGFBR2
Receptor
JUN MYC NFKB1 RELA STAT1 STAT2 STAT5A ESR1 SP1 FOXP3 SMAD9 E2F4
TF
CD86 GRN TNFSF13
Target
D
Monocyte and CD8+ IRhigh T cell
CD86 GRN TNFSF13
Ligand
CD28 CTLA4 TNFRSF1A
Receptor
NFATC1 NFATC2 NFATC3
TF
B
Leukemia-like cell and monocyte
GRN LGALS9 MIF TGFB1
Ligand
SORT1 TNFRSF1A TNFRSF1B HAVCR2 PTPRC CXCR2 CXCR4 ENG TGFBR1 TGFBR2
Receptor
ESR1 JUN MYC NFKB1 RELA SP1 STAT1 STAT5A STAT2 FOXP3 SMAD9 E2F4
TF
CD86 TNFSF13 GRN
Target
C
Monocyte and T cell
HLA-E - CD94:NKG2E
HLA-E - CD94:NKG2C
HLA-E - CD94:NKG2A
HLA-E - KLRK1
HLA-A - KIR3DL1
HLA-E - KLRC2
HLA-E - KLRC1
HLA-B - KIR3DL2
HLA-F - KIR3DL2
HLA-F - KIR3DL1
HLA-C - KIR2DL3
HLA-F - CD8B
HLA-E - CD8B
HLA-C - CD8B
HLA-B - CD8B
HLA-A - CD8B
HLA-F - CD8A
HLA-E - CD8A
HLA-C - CD8A
HLA-B - CD8A
HLA-A - CD8A
CD86 - CTLA4
CD86 - CD28
CD48 - CD244A
THBS1 - CD47
LGALS9 - HAVCR2
LGALS9 - CD45
GRN - SORT1
TNFSF13 - TNFRSF13B
TNFSF13 - TNFRSF17
monocyte -> CD4 Tm
monocyte -> CD4 Teff
monocyte -> CD4 IRhigh T
monocyte -> CD8 Tm
monocyte -> CD8 Teff
monocyte -> CD8 IRhigh T
monocyte -> NKT
monocyte -> NKT IRhigh
Commun. Prob.
max
min
p-value
0.01 < p < 0.05
p < 0.01
E
Leukemia-like cell and DC
APP CD99 F11R GRN HLA-DMA HLA-DRA HLA-DRB1 ICAM1 ICAM2 IL16 IL1B LGALS9 MDK MIF OSM PTPRC SEMA4D TGFB1
Ligand
NCSTN PILRA F11R ITGAL SORT1 TNFRSF1A TNFRSF1B CD4 CCR5 IL1R1 IL1R2 IL1RAP PTPRC ITGB1 NOTCH2 SDC4 CXCR4 IL6ST MRC1 PLXNB2 ENG ITGAV
Receptor
JUN NFKB1 RELA STAT1 STAT5A ESR1 FOXP3
TF
CD86 GRN
Target
H
DC and CD8+ IRhigh T cell
CD86 GRN
Ligand
CD28 CTLA4 TNFRSF1A
Receptor
NFATC1 NFATC2 NFATC3
TF
F
Leukemia-like cell and DC
GRN LGALS9 MIF TGFB1
Ligand
SORT1 TNFRSF1A TNFRSF1B PTPRC CXCR4 ENG ITGAV
Receptor
ESR1 JUN NFKB1 RELA STAT1 STAT5A FOXP3
TF
CD86 GRN
Target
G
DC and T cell
HLA-E - CD94:NKG2E
HLA-E - CD94:NKG2C
HLA-E - CD94:NKG2A
HLA-E - KLRK1
HLA-A - KIR3DL1
HLA-E - KLRC2
HLA-E - KLRC1
HLA-B - KIR3DL2
HLA-F - KIR3DL2
HLA-F - KIR3DL1
HLA-C - KIR2DL3
HLA-F - CD8B
HLA-E - CD8B
HLA-C - CD8B
HLA-B - CD8B
HLA-A - CD8B
HLA-F - CD8A
HLA-E - CD8A
HLA-C - CD8A
HLA-B - CD8A
HLA-A - CD8A
CD86 - CTLA4
CD86 - CD28
CD48 - CD244A
THBS1 - CD47
LGALS9 - CD44
LGALS9 - HAVCR2
LGALS9 - CD45
GRN - SORT1
TNFSF13 - TNFRSF13B
TNFSF13 - TNFRSF17
MIF - (CD74+CXCR4)
HBEGF - (ERBB2+ERBB4)
HBEGF - ERBB4
HBEGF - (EGFR+ERBB2)
HBEGF - EGFR
TGFB1 - (ACVR1+TGFBR1)
TGFB1 - (TGFBR1+TGFBR2)
DC -> CD4 Tm
DC -> CD4 Teff
DC -> CD4 IRhigh T
DC -> CD8 Tm
DC -> CD8 Teff
DC -> CD8 IRhigh T
DC -> NKT
DC -> NKT IRhigh
Commun. Prob.
max
min
p-value
0.01 < p < 0.05
p < 0.01

Figure S10. Interactions among leukemia-like cells, monocytes, DCs, and T cells in post-SCT relapse AML patients.

A The molecular regulatory network between leukemia-like cells and monocyte. The multilayer network contained four layers of ligand, receptor, TF, and target, which were marked by different colors. The targets were the intersection of ligands involved in the interaction between monocyte and $CD8^+$ T_{ex}.

B The molecular regulatory network between leukemia-like cells and monocyte. The multilayer network contained four layers of ligand, receptor, TF, and target, which were marked by different colors. The ligands were the intersection of ligands involved in the interaction between leukemia-like cells and $CD8^+$ T_{ex} in pre-SCT NR settings, and the targets were the intersection of ligands involved in the interaction between monocyte and $CD8^+$ T_{ex}.

C Dot plot showing the intersections of significant functional ligand-receptor pairs that contribute to the signaling sending from monocyte to eight T cell subtypes.

D The molecular regulatory network between monocyte and $CD8^+$ T_{ex}. The multilayer network contained three layers of ligand, receptor, and TF, which were marked by different colors. The ligands were the intersection of targets involved in the interaction between leukemia-like cells and monocyte.

E The molecular regulatory network between leukemia-like cells and DC. The multilayer network contained four layers of ligand, receptor, TF, and target, which were marked by different colors. The targets were the intersection of ligands involved in the interaction between DC and $CD8^+$ T_{ex}.

F The molecular regulatory network between leukemia-like cells and DC. The multilayer network contained four layers of ligand, receptor, TF, and target, which were marked by different colors. The ligands were the intersection of ligands involved in the interaction between leukemia-like cells and $CD8^+$ T_{ex} in pre-SCT NR settings, and the targets were the intersection of ligands involved in the interaction between DC and $CD8^+$ T_{ex}.

G Dot plot showing the intersections of significant functional ligand-receptor pairs that contribute to the signaling sending from DC to eight T cell subtypes.

H The molecular regulatory network between DC and $CD8^+$ T_{ex}. The multilayer network contained three layers of ligand, receptor, and TF, which were marked by different colors. The ligands were the intersection of targets involved in the interaction between leukemia-like cells and DC.

For dot plots (**C** and **G**), dot color represents communication probabilities, dot size represents one-sided permutation test *p*-values, and empty space indicates that the communication probability is zero.

For molecular regulatory network plots (**A**, **B**, **D**, **E**, **F**, and **H**), the ligands were the intersection between CellChat and scMLnet analysis.
